# Linking genetic determinants with salinity tolerance and ion relationships in eggplant, tomato and pepper

**DOI:** 10.1038/s41598-021-95506-5

**Published:** 2021-08-11

**Authors:** Donald L. Suarez, Nydia Celis, Jorge F. S. Ferreira, Trevor Reynolds, Devinder Sandhu

**Affiliations:** 1 USDA-ARS, U.S. Salinity Lab, 450 W Big Springs Road, Riverside, CA 92507 USA; 2 College of Natural and Agricultural Sciences, University of California Riverside, 900 University Avenue, Riverside, CA 92521 USA

**Keywords:** Plant sciences, Environmental sciences

## Abstract

The Solanaceae family includes commercially important vegetable crops characterized by their relative sensitivity to salinity. Evaluation of 8 eggplant (*Solanum melongena*), 7 tomato (*Solanum lycopersicum*), and 8 pepper (*Capsicum* spp.) heirloom cultivars from different geographic regions revealed significant variation in salt tolerance. Relative fruit yield under salt treatment varied from 52 to 114% for eggplant, 56 to 84% for tomato, and 52 to 99% for pepper. Cultivars from all three crops, except Habanero peppers, restricted Na transport from roots to shoots under salinity. The high salt tolerance level showed a strong association with low leaf Na concentration. Additionally, the leaf K-salinity/K-control ratio was critical in determining the salinity tolerance of a genotype. Differences in relative yield under salinity were regulated by several component traits, which was consistent with the gene expression of relevant genes. Gene expression analyses using 12 genes associated with salt tolerance showed that, for eggplant and pepper, Na^+^ exclusion was a vital component trait, while sequestration of Na^+^ into vacuoles was critical for tomato plants. The high variability for salt tolerance found in heirloom cultivars helped characterize genotypes based on component traits of salt tolerance and will enable breeders to increase the salt tolerance of Solanaceae cultivars.

## Introduction

Agriculture is responsible for over 80% of the water consumption in the United States (https://www.usda.gov/media/blog/2021/02/11/usda-invests-data-agricultural-irrigation-improvements). Sufficient water is vital for food security, however, groundwater aquifers worldwide are being overused^1^. This overuse threatens water security, welfare, and population survival^2,3^. Therefore, sustainability of agriculture, mainly in semi-arid regions, is highly dependent on alternative water resources, which are generally higher in salinity than freshwater. Thus, salinity is an increasing threat to vegetable production in arid and semi-arid regions.

Over the last 50 years, there has been a steady reduction in the biodiversity of vegetables and other crops due to monoculture^4^. New varieties have been bred for increased yield, better appearance, and tolerance to pests and diseases, with little emphasis given to their resilience to major abiotic stresses such as drought and salinity. Such an approach reduces the number of cultivars upon which our global food security depends and does not consider the need for a broad genetic pool, which is needed to develop new varieties resilient to abiotic stresses imposed by climatic change. Solanaceae is a family of major crops, from which at least 15 genera are grown to provide food worldwide^4^. In 2010, 28 million hectares of solanaceous food crops were cultivated globally, producing around 540 million tons of potatoes, tomatoes, eggplants, and *Capsicum* spp. combined^4^. Extensive differences in salt tolerance have been reported among Solanaceae species. Huckleberry (*Solanum scabrum* Mill.) total leaf dry weight and leaf area were reduced by the application of 50 mM NaCl in 25 and 18% while these reductions were 47 and 55% in eggplant (*Solanum melongena* L.), respectively^5^. The biomass and leaf area reductions were attributed to greater Na concentration in eggplant leaves as compared to huckleberry. Solanaceae is a large tropical family with members characterized by their relative sensitivity to salinity^6^. Losses in fruit yield start at EC values of the soil saturation extract (EC_e_) of 1.1 dS m^−1^ for eggplant, 2.5 dS m^−1^ for tomato, and 1.5 dS m^−1^ for pepper^6^. The percent-fruit-yield decline per unit increase in salinity (EC_e_) was reported at 6.9, 9.9, and 14 for eggplant, tomato, and pepper, respectively^6^. These results suggest that at higher salinity, eggplant exhibits greater salt tolerance than tomato. However, eggplant and tomato had comparable decreases in vegetative yield under salinity of 3.7 dS m^−1^ when ‘Black Bell’ eggplant and ‘Ikram’ tomato plants were grafted onto two tomato rootstocks^7^. The reported salt-tolerance values for several crop species were often based on a relatively small number of studies (sometimes just one) where soil salinity was characterized and several salt levels were applied. However, cultivar differences, in general, were not reported in these databases.

It is well known that cultivar differences provide significant genetic variation for crop improvement^8^. The evaluation of four eggplant cultivars under irrigation water EC (EC_iw_) ranging from 1.2 to 5.2 dS m^−1^ showed large differences in shoot biomass accumulation and for the quantum yield of photosystem II between salt-tolerant and salt-sensitive cultivars^9^. After 25 days under EC_iw_ = 5.2 dS m^−1^, fresh biomass decreased by 87% and 88% in ‘Adriatica’ and ‘Black Beauty’, respectively. In contrast, under the same salinity, fresh biomass decrease was only 37% and 36% for ‘Bonica’ and ‘Galine’, respectively^9^. The salt-tolerance threshold level (the level at which yield first starts to decrease) was different for fruit yield and shoot biomass in eggplant^10,11^. The threshold was between EC_e_ = 1.1 and 1.5 dS m^−1^ for fruit yield^10^, whereas it was 6.7 dS m^−1^ for shoot biomass^11^. In a study with two eggplant cultivars, ‘Bemisal’ produced greater fresh weight than ‘Dilnasheen’, but both cultivars had approximately 50% reduction in fruit yield under 100 mM NaCl (EC_iw_ of 10 dS m^−1^)^12^. Sensitivity to Na was attributed to the inability of eggplant to restrict Na uptake and transport to the leaf^12^. The literature is rich in papers dealing with strategies to mitigate salinity stress in eggplant, but we could not find many publications evaluating different cultivars for fruit yield under field conditions.

Tomato plants have been classified as ‘moderately sensitive’ to salinity, tolerating EC_e_ = 2.5 dS m^−1^ without fruit yield reduction^6^. However, in a two-year experiment in California, tomato fresh fruit yield was not significantly reduced when irrigated with EC_iw_ = 8.1 dS m^−1^^13^. Greenhouse-cultivated tomato plants irrigated with EC_iw_ = 7.5 dS m^−1^ (moderate salinity) grew significantly better when pre-conditioned with a saline solution of 10 mM NaCl (EC_iw_ ~ 1.0 dS m^−1^)^14^. In another study, the salt tolerance differences among tomato (*Solanum lycopersicum* L.) cultivars were directly correlated with their K^+^ and Ca^2+^ absorption^15^. Thus, it seems that tomato soil salinity threshold varies according to the cultivar used, and breeding of salt-tolerant cultivars will require exploring different mechanisms that play important roles during salinity stress^16^.

Pepper cultivation may differ from that of tomato and eggplant as the objective is not always the production of large, sweet fruits. Peppers are also cultivated to produce fruits with a pungency (heat in peppers provided by capsaicin alkaloids) for culinary purposes or even for their potential use as a source of capsaicin and dihydrocapsaicin as raw materials for topical anti-inflammatory and anti-rheumatoid arthritis medicines^17,18^.

A recent study reported differences in salt tolerance of two pepper cultivars under salinities ranging from 0 to 120 mM NaCl^19^. These authors reported that the superior salt tolerance of ‘Quadrato D’Asti’ pepper plants, compared to ‘Cazzone Giallo’, was due to their more efficient exclusion of Cl from shoots (leaves + stems). The accumulation of Na in both cultivars was 7.6 (‘Quadrato D’Asti’) to 12.5-fold (‘Cazzone Giallo’) smaller than Cl^19^. The authors did not discriminate Na or Cl accumulation between roots and shoots but, judging from the small accumulation of Na in shoots of both cultivars (11–12 mg kg^−1^ at 120 mM NaCl), most Na must have been retained by roots. Interestingly, although both cultivars accumulated similar concentrations of both Na and Cl in shoots at 30 mM NaCl, ‘Quadrado D’Asti’ had no fruit yield decrease at that salt concentration^19^. The greatest significant fruit yield loss was reported in plants irrigated with the NaCl solution, followed by KCl, and the lowest loss with the K_2_SO_4_ solution, probably due to the accumulation of Cl, not Na, in shoots^20^. Pepper salt tolerance was improved by grafting pepper plants onto rootstocks that restricted both Na and Cl transport to shoots and improved ion selectivity, such as preferred accumulation of K versus Na in shoots^21^.

Studies evaluating the relative importance of Na or Cl, especially in vegetable plants, are limited. In both tomato^22^ and eggplant^23^, there was no significant difference in yield between salt solutions dominant in Cl^−^ or SO_4_^2−^, suggesting that Na^+^ is the dominant toxic ion for these species. This conclusion was confirmed by predicting eggplant yield of two different salt-tolerant cultivars, based on leaf Na concentration, but not with leaf Cl concentration^23^.

Effective comparison of crop salt tolerance among studies is difficult as experimental protocols do not have standardized parameters, the experiments rarely include the same cultivars, and do not contain enough (or the same) salt levels to allow for interexperiment comparisons. Among these experimental parameters are different temperatures, with plants under cooler temperatures exhibiting greater salt tolerance^24^; the salt composition of saline solutions^25^; outdoor versus greenhouse conditions, compounding of abiotic stresses such as drought and salinity^26^; differences in developmental stages^27^ and differences in plant organ yield (vegetative biomass versus fruit yield^11^. Another difficulty in comparing results among studies involves the reporting of soil extract salinity (EC_e_) or irrigation water salinity (EC_iw_). The latter is difficult to convert to EC_e_ unless the leaching fraction or water budget data, including irrigation volumes, is available.

Heirloom cultivars have been grown for over 50 years and are appreciated for their unique appearance, flavor, cultural and ethnic significance, and their role in sustainable food production by small-scale farmers^28^. These authors also remarked on the urgent need to explore heirloom cultivars for their yield, organoleptic characteristics, and stress tolerance. The large genetic pool provided by heirloom cultivars for tolerance to abiotic stresses should assist in the development of more salt-tolerant vegetables.

Several genes involved in salinity response are known in plants^29,30^. Transporter proteins are critical in regulating Na and Cl concentrations in plants, and various genes involved in these processes have been characterized^29,31^. Some studies have focused on identifying salt tolerance genes in Solanaceae. The differences in relative salt tolerance of two pepper cultivars were characterized by the more salt-tolerant cultivar having slightly increased K/Na shoot ratio, decreased K/Na root ratio, thus greater K leaf/root ratio and lower Na leaf/root ratio^32^. The enhanced performance of the salt-tolerant pepper cultivar was explained by the upregulation (or overexpression) of *AKT1*, *KAT1,* and *SOS1* genes in the leaves. Similarly, overexpression of *SlSOS2* in transgenic tomato resulted in enhanced salt tolerance due to Na^+^ exclusion from roots^33^. The superior salt tolerance of huckleberry as compared to eggplant was attributed to greater overexpression of *SsHKT* in huckleberry compared to eggplant under salt stress^34^. Silencing the *CaSBP12* gene in chili pepper (*C. annuum*. L.) increased salt tolerance measured from vegetative biomass and decreased shoot Na^35^. Similarly, silencing the *SINAC11* gene reduced salinity and drought tolerance of tomato as evidenced by germination rate, chlorophyll content, and malondialdehyde (MDA) content^36^. Despite some studies on salinity tolerance, the understanding of genetic networks involved in salinity tolerance is still limited in Solanaceae. Understanding different genetic factors involved in salinity stress will be critical in developing salt-tolerant genotypes in Solanaceae.

Our objectives were to (1) examine the salt tolerance of 23 Solanaceae cultivars (eight eggplant, seven tomato, and eight pepper) from different geographic regions, expressed as relative fruit yield over the life cycle of the plant under field conditions, (2) study the relationship between fruit yield and tissue ion composition, and (3) recognize genetic components regulating salinity tolerance in the Solanaceae family. Previous studies of Solanaceae salt tolerance have examined the relation of germination and shoot growth to gene expression for a few genotypes. In our attempt to maximize salt tolerance variability with commercially relevant genotypes, we selected only heirloom cultivars of three species from a worldwide range of geographic locations and climates.

## Results

### Fruit yields in response to salinity

#### Eggplant (*Solanum melongena* L.)

**Figure 1:**
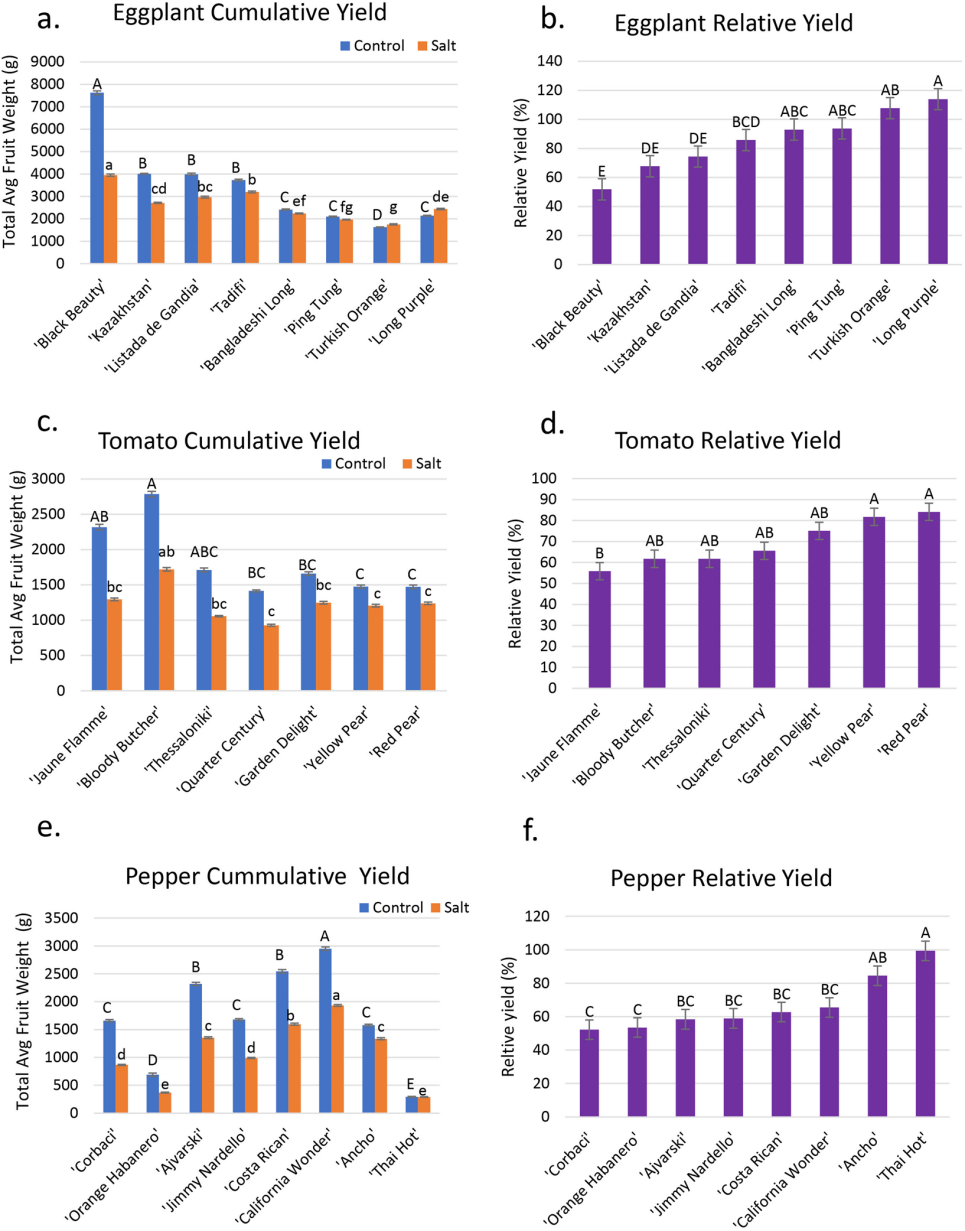
Fruit yield of eggplant, tomato, and pepper under control and saline irrigation waters. (**a**) Cumulative fresh weight fruit yield for 8 eggplant cultivars. (**b**) Relative yield (yield of saline treatment/yield of control) for 8 eggplant cultivars, expressed as a percent. (**c**) Cumulative fresh weight fruit yield for 7 tomato cultivars. (**d**) Relative yield for 7 tomato cultivars, expressed as a percent. (**e**) Cumulative fresh weight fruit yield for 8 pepper cultivars. (**f**) Relative yield for 8 pepper cultivars, expressed as a percent. Capital letters denote significant differences in yield under control conditions, while lowercase letters denote significant yield differences under saline conditions (*p* < 0.05). Error bars represent standard errors.

Under control irrigation water (EC_iw_ = 0.65 dS m^−1^), the highest cumulative fruit yield per plant was recorded for ‘Black Beauty’ (7624 g), and the lowest for ‘Turkish Orange’ (1627 g) (Fig. 1a). Under salinity conditions (EC_iw_ = 4.0 dS m^−1^), ‘Black Beauty’ (3950 g) was also the highest fruit-yielding cultivar, significantly outproducing all other cultivars, while ‘Turkish Orange’ (1753 g) and ‘Ping Tung’ (1968 g) were the lowest (Fig. 1a).

The relative yield of the marketable part of the plant (yield under saline treatment/yield under control) is the commonly used criterion for evaluating salt tolerance^6^. Based on relative fruit yield data, ‘Black Beauty’ and ‘Kazakhstan’ were salt-sensitive, with a relative yield of 52% and 68% when exposed to salinity (Fig. 1b), while ‘Long Purple’ (114%) and ‘Turkish Orange’ (108%) were the most salt-tolerant cultivars with no cumulative fruit yield loss under saline treatment (Fig. 1b).

#### Tomato (*Solanum lycopersicum* L.)

Under control conditions, cumulative fruit yield was significantly greater for ‘Bloody Butcher’ (2785 g), ‘Jaune Flamme’ (2317 g), and ‘Thessaloniki’ (1,712 g), with ‘Bloody Butcher’ having the greatest cumulative yield and ‘Quarter Century’ (1415 g) the lowest (Fig. 1c). The remaining cultivars produced significantly lower cumulative fruit yields than ‘Bloody Butcher’, but there were no significant differences among them (Fig. 1c). Under saline conditions, ‘Quarter Century’ had the lowest cumulative yield (928 g), and ‘Bloody Butcher’ (1719 g) the highest. Although ‘Jaune Flamme’ produced a heavier crop than both ‘Red Pear’ and ‘Yellow Pear’ under control conditions, these three cultivars had similar fruit yields under salinity (Fig. 1c). The greatest relative yield was for ‘Red Pear’ (84%), closely followed by ‘Yellow Pear’ (82%), then ‘Garden delight’ (75%) while the lowest relative yield (56%) was for ‘Jaune Flamme’ (Fig. 1d).

#### Pepper (*Capsicum* spp.)

Under control conditions, the highest cumulative fruit yield was achieved by ‘California Wonder’ (2951 g), which was significantly greater than all other cultivars, followed by ‘Costa Rican’ (2545 g), ‘Ajvarski’ (2318 g), ‘Corbaci’ similar to ‘Jimmy Nardello’, ‘Orange Habanero’, and ‘Thai Hot’ (Fig. 1e). The lowest yield was for ‘Thai Hot’ (291 g), which produced numerous red peppers of very small size, and maintained its cumulative fruit yield under salinity (Fig. 1e). Under saline conditions, the significantly greatest cumulative yield was also for ‘California Wonder’ (1932 g), followed by ‘Costa Rican’ (1597 g), ‘Ajvarski’ (1353 g) similar to ‘Ancho’ (1335 g), ‘Jimmy Nardello’ (989 g) similar to ‘Corbaci’ (866 g), and ‘Orange Habanero’ pepper (369 g) similar to ‘Thai Hot’ (290 g) (Fig. 1e).

The highest relative yield was for ‘Thai Hot’ (100%) as it had no yield loss, followed by ‘Ancho’ (85%) (Fig. 1f). The other cultivars had relative fruit yields ranging from 52% (‘Cobaci’) to 65% (‘California Wonder’).

### Tissue ion concentrations in response to salinity

#### Eggplant

**Figure 2:**
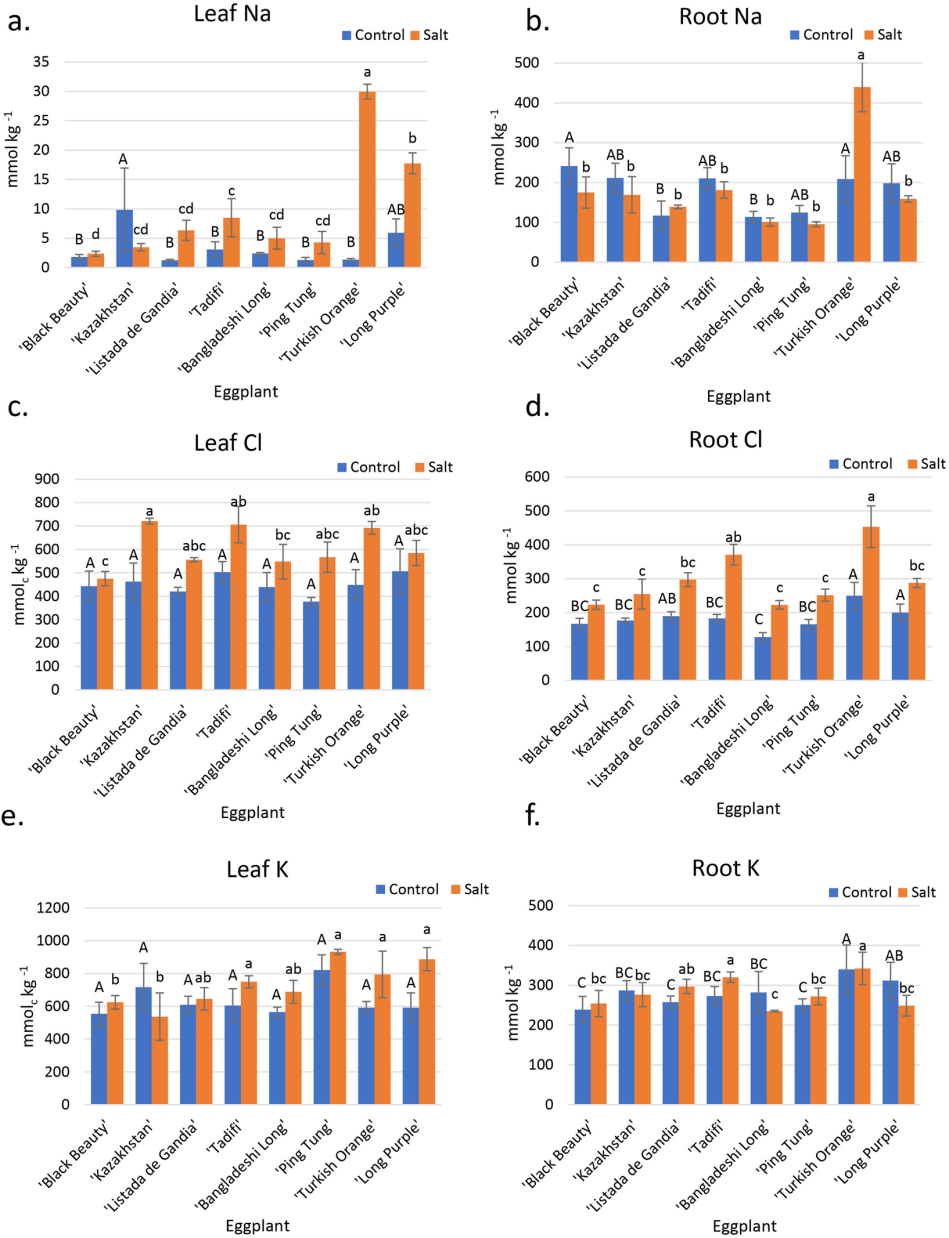
Tissue ion concentrations of eight eggplant cultivars irrigated with control and saline irrigation waters. (**a**) Leaf Na concentrations. (**b**) Root Na concentrations. (**c**) Leaf Cl concentrations. (**d**) Root Cl concentrations. (**e**) Leaf K concentrations. (**f**) Root K concentrations. Capital letters denote differences among cultivars under control conditions, while lowercase letters denote significant differences under saline conditions (*p* < 0.05). Error bars represent standard errors.

This species showed significant increases in leaf Na concentration between control and salt treatments for all cultivars, except for ‘Black Beauty’ (Fig. 2a). There were also significant differences among cultivars. Under saline conditions, ‘Turkish Orange’ (30 mmol kg^−1^) accumulated the most Na in the leaves, followed by ‘Long Purple’ (17.7 mmol kg^−1^), and ‘Tadifi’ (8.5 mmol kg^−1^). ‘Black Beauty’ accumulated the least concentration of Na (2.4 mmol kg^−1^) in the leaves, followed by ‘Kazakhstan’ (3.5 mmol kg^−1^) and ‘Ping Tung’ (4.3 mmol kg^−1^) (Fig. 2a). In terms of increase relative to the control, ‘Turkish Orange’ had a 2070% Na increase in the leaves, ‘Long Purple’ a 198% increase, and ‘Black Beauty’ a 31% increase (Fig. 2a). ‘Turkish Orange’ accumulated significantly more Na in the roots (439 mmol kg^−1^) than all other cultivars under the saline treatment (Fig. 2b).

The Cl leaf concentration data showed no significant differences among varieties under control conditions, with concentrations ranging from 377 mmol kg^−1^ for ‘Ping Tung’ to 507 mmol kg^−1^ for ‘Long Purple’ (Fig. 2c). However, most varieties had increases in leaf Cl under the saline treatment with significant differences in leaf Cl among cultivars (Fig. 2c). ‘Kazakhstan’ (721 mmol kg^−1^), ‘Tadifi’ (706 mmol kg^−1^), and ‘Turkish Orange’ (692 mmol kg^−1^) accumulated the most Cl in their leaves. ‘Black Beauty’ (475 mmol kg^−1^) accumulated the least amount of Cl in the leaves, followed by ‘Bangladeshi Long’ (548 mmol kg^−1^) (Fig. 2c). ‘Black Beauty’ showed only a slight increase in leaf Cl under salt treatment (475 mmol kg^−1^) as compared to control (443 mmol kg^−1^). ‘Turkish Orange’ accumulated significantly more Cl (453 mmol kg^−1^) in the roots than all other cultivars under salt treatment (Fig. 2d).

There were significant differences in K concentrations both between treatments and among cultivars, but no significant interaction between treatments and cultivars (Fig. 2e). Under control conditions, there were no significant differences in leaf K among cultivars, with values ranging from 592 to 717 mmol kg^−1^. Under salinity, ‘Ping Tung’ (932 mmol kg^−1^), ‘Long Purple’ (887 mmol kg^−1^), and ‘Turkish Orange’ (794 mmol kg^−1^), accumulated the most K in the leaves. ‘Kazakhstan’ (537 mmol kg^−1^) and ‘Black Beauty’ (625 mmol kg^−1^) accumulated the least amount of K in their leaves (Fig. 2e). Under salinity, ‘Turkish Orange’ (342 mmol kg^−1^) and ‘Tadifi’ (320 mmol kg^−1^) accumulated significantly more K in the roots than all other cultivars, and ‘Bangladeshi Long’ (235 mmol kg^−1^) accumulated the least K under the salt treatment (Fig. 2f). Eggplant cultivars varied significantly in their relative leaf K concentration under salinity compared to control (K-salinity/K-control). The two most salt-tolerant cultivars of eggplant, ‘Long Purple’ and ‘Turkish Orange’, had leaf K-salinity/K-control ratios of 1.43 and 1.37, respectively (K data in Fig. 2e). However, even the two most salt-sensitive varieties, ‘Black Beauty’ and ‘Kazakhstan’, had K-salinity/K-control ratios of 0.98 each.

There were no significant differences in leaf Ca concentration among treatments or for the interaction between Ca and salinity, although there were significant differences among cultivars (Supplementary Fig. S1a). ‘Ping Tung’ (590 mmol kg^−1^) accumulated the most Ca in the roots under control, significantly greater than all, except for ‘Bangladeshi Long’ (507 mmol kg^−1^). Under salinity, ‘Bangladeshi Long’ (474 mmol kg^−1^) had the greatest Ca concentration, along with ‘Ping Tung’ (469 mmol kg^−1^), significantly greater Ca for all but one cultivar (‘Tadifi’) (426 mmol kg^−1^), while Black Beauty’ (220 mmol kg^−1^) and ‘Turkish Orange’ (242 mmol kg^−1^) had the least Ca concentrations (Supplementary Fig. S1b).

There were no significant differences in leaf Mg concentrations among cultivars under control conditions (Supplementary Fig. S2a). Leaf Mg increased under saline treatment for all cultivars except ‘Long Purple’. Under salinity, ‘Turkish Orange’ (356 mmol kg^−1^) and ‘Tadifi’ (354 mmol kg^−1^) accumulated the most Mg in the leaves while ‘Long Purple’ (232 mmol kg^−1^) accumulated the least amount, significantly less than ‘Turkish Orange’ (Supplementary Fig. S2a). ‘Turkish Orange’ (198 mmol kg^−1^) accumulated significantly more Mg in the roots than the other varieties under both control and salinity conditions (Supplementary Fig. S2b).

There were no significant differences in leaf nitrogen concentration between salinity treatments or between the interaction of the two, but there were significant differences among cultivars. Under salinity, ‘Turkish Orange’ (4%) and ‘Kazakhstan’ (3%) accumulated the most nitrogen in the leaves (Supplementary Fig. S3a).

#### Tomato

**Figure 3:**
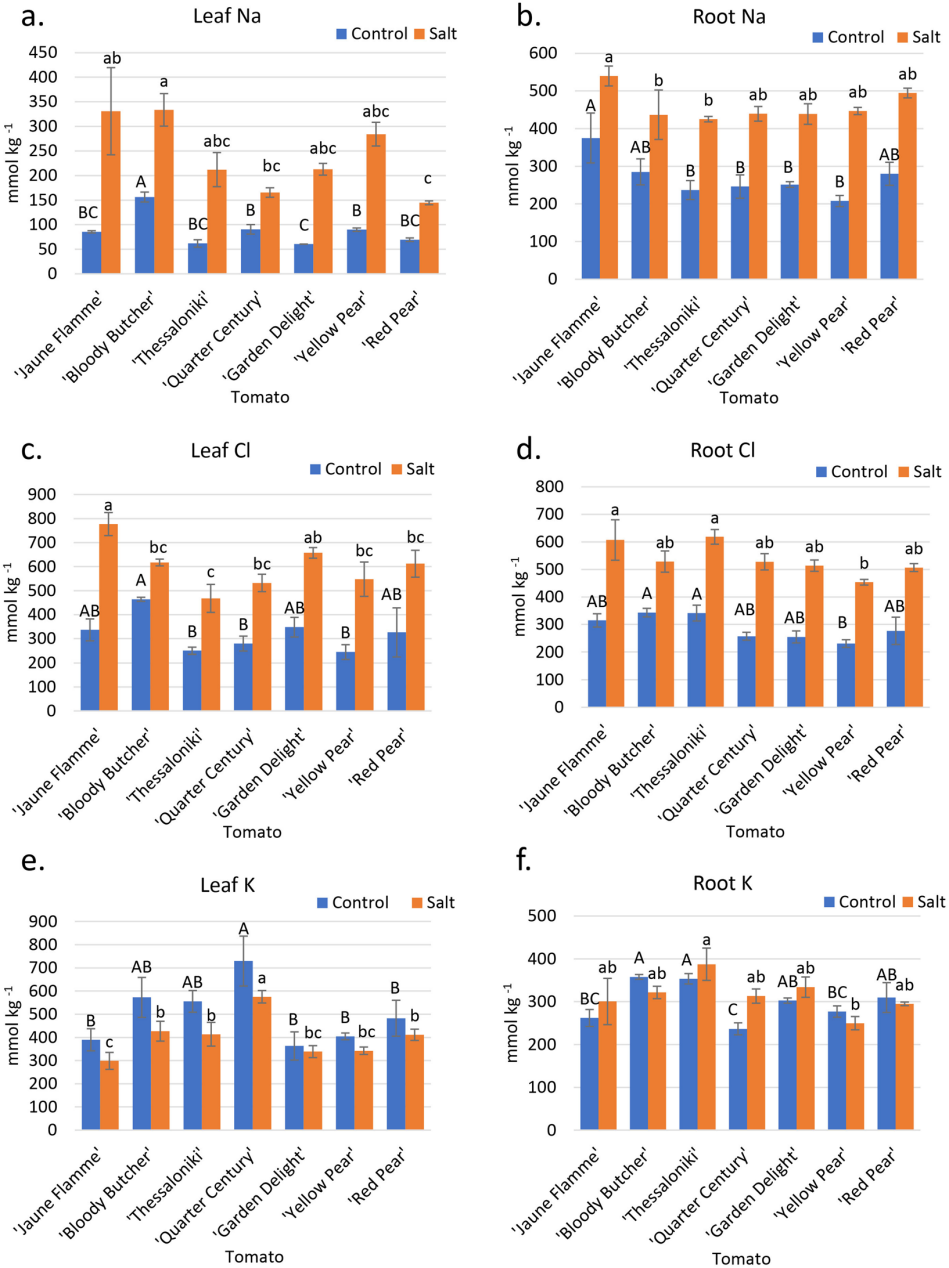
Tissue ion concentrations of seven tomato cultivars irrigated with control and saline irrigation waters. (**a**) Leaf Na concentrations. (**b**) Root Na concentrations. (**c**) Leaf Cl concentrations. (**d**) Root Cl concentrations. (**e**) Leaf K concentrations. (**f**) Root K concentrations. Capital letters denote differences among cultivars under control conditions, while lowercase letters denote significant differences under saline conditions (*p* < 0.05). Error bars represent standard errors.

Under control conditions, ‘Bloody Butcher’ accumulated significantly more Na (156 mmol kg^−1^) in its leaves than all other cultivars, followed by ‘Quarter Century’ (90 mmol kg^−1^) and ‘Yellow Pear’ (90 mmol kg^−1^) (Fig. 3a). ‘Garden Delight’ accumulated the least amount of Na (60 mmol kg^−1^) in the leaves, significantly less than the three highest Na accumulators. Under saline conditions, ‘Bloody Butcher’ (334 mmol kg^−1^), followed by ‘Jaune Flamme’ (331 mmol kg^−1^) accumulated the most leaf Na, while ‘Red Pear’ (145 mmol kg^−1^) accumulated the lowest concentration of Na, followed by ‘Quarter Century’ (165 mmol kg^−1^). Compared to other cultivars, ‘Jaune Flamme’ accumulated the most Na in the roots under both control (375 mmol kg^−1^) and saline (540 mmol kg^−1^) conditions (Fig. 3b).

Under control conditions, ‘Bloody Butcher’ (464 mmol kg^−1^) accumulated the most Cl, significantly greater than the lowest accumulators, ‘Yellow Pear’ (245 mmol kg^−1^) ‘Quarter ‘Thessaloniki’ (250 mmol kg^−1^), and Century’ (280 mmol kg^−1^), (Fig. 3c). Under salinity, ‘Jaune Flamme’ (777 mmol kg^−1^) accumulated the most Cl in the leaves, followed by ‘Garden Delight’ (657 mmol kg^−1^). ‘Thessaloniki’ (468 mmol kg^−1^) accumulated the least amount of Cl in the leaves, significantly less than the top Cl accumulators (Fig. 3c). However, under salinity, ‘Thessaloniki’ (618 mmol kg^−1^) was the top root Cl accumulator, and ‘Yellow Pear’ (453 mmol kg^−1^) was the least Cl accumulator (Fig. 3d).

‘Quarter Century’ (730 mmol kg^−1^) accumulated significantly more K in the leaves than all other cultivars except ‘Bloody Butcher’ (573 mmol kg^−1^) and ‘Thessaloniki’ (556 mmol kg^−1^) under control conditions (Fig. 3e). Under saline conditions, K decreased for all cultivars, but ‘Quarter Century’ (575 mmol kg^−1^) had significantly greater K than all other cultivars, and ‘Jaune Flamme’ (299 mmol kg^−1^) accumulated the least amount of K, significantly less than all cultivars except ‘Garden Delight’ (339 mmol kg^−1^) and ‘Yellow Pear’ (342 mmol kg^−1^) (Fig. 3e). ‘Thessaloniki’ (387 mmol kg^−1^) was the top K accumulator in roots under salinity, and ‘Yellow Pear’ (250 mmol kg^−1^) was the least K accumulator (Fig. 3f). ‘Red Pear’ and ‘Yellow Pear’ (the two most salt-tolerant cultivars) had K-salinity/K-control ratios of 0.85 each, and ‘Jaune Flamme’ and ‘Bloody Butcher’ (the two most salt-sensitive cultivars) had K-salinity/K-control ratios of 0.68 and 0.75, respectively (K data in Fig. 3e).

Leaf concentrations of Ca were significantly greater in ‘Garden Delight’ (1,110 mmol kg^−1^) and ‘Red Pear’ (980 mmol kg^−1^) as compared to ‘Thessaloniki’ (652 mmol kg^−1^) and ‘Quarter Century’ (691 mmol kg^−1^) under control conditions. Under salt conditions, ‘Garden Delight’ (933 mmol kg^−1^) accumulated significantly more Ca in the leaves than ‘Yellow Pear’ (733 mmol kg^−1^), ‘Red Pear’ (692 mmol kg^−1^), ‘Quarter Century’ (643 mmol kg^−1^), and ‘Thessaloniki’ (619 mmol kg^−1^) (Supplementary Fig. S1c). ‘Red Pear’ (512 mmol kg^−1^) accumulated significantly more Ca in the roots than ‘Yellow Pear’ (339 mmol kg^−1^) under saline treatment (Supplementary Fig. S1d).

Under control conditions, ‘Red Pear’ (346 mmol kg^−1^) had the greatest leaf Mg concentrations, significantly different than the lowest accumulator, ‘Thessaloniki’ (230 mmol kg^−1^), while under saline conditions, ‘Red Pear’ (418 mmol kg^−1^), ‘Yellow Pear’ (394 mmol kg^−1^) and ‘Garden Delight’ (389 mmol kg^−1^) had significantly greater Mg than ‘Thessaloniki’ (280 mmol kg^−1^) (Supplementary Fig S2c). The Mg concentration was greater in the salt treatment than in the control. ‘Red Pear’ (192 mmol kg^−1^) accumulated significantly more Mg in the roots than ‘Bloody Butcher’ (118 mmol kg^−1^) and ‘Quarter Century’ (120 mmol kg^−1^) under the saline treatment (Supplementary Fig S2d).

There were no significant differences in N leaf concentration under control, but ‘Red Pear’ (4%), ‘Yellow Pear’(3%), and ‘Jaune Flamme’ (3%) accumulated significantly more N in the leaves than ‘Quarter Century’ (2.6%) under saline conditions (Supplementary Fig. S3c).

#### Pepper

**Figure 4:**
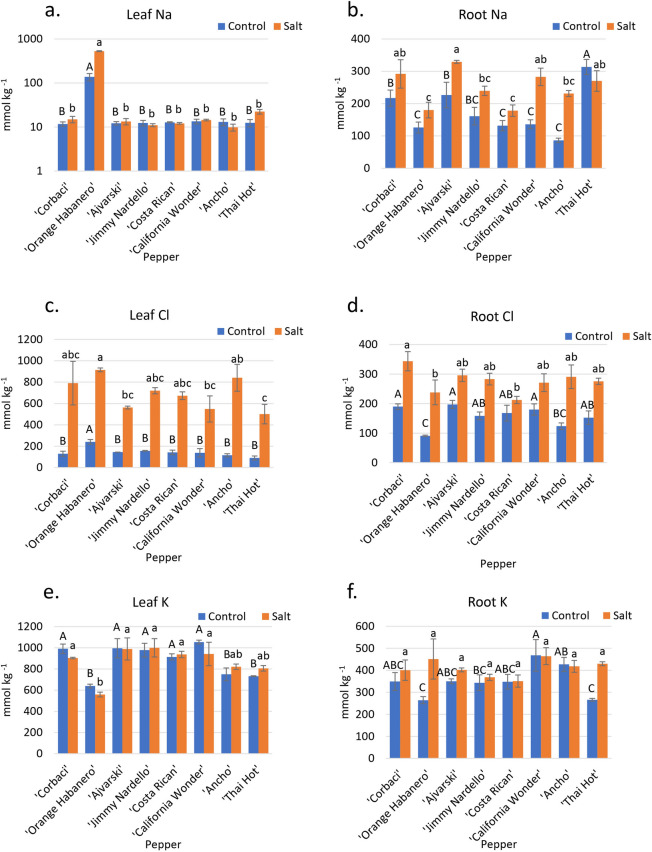
Tissue ion concentrations of eight pepper cultivars irrigated with control and saline irrigation waters. (**a**) Leaf Na concentrations. (**b**) Root Na concentrations. (**c**) Leaf Cl concentrations. (**d**) Root Cl concentrations. (**e**) Leaf K concentrations. (**f**) Root K concentrations. Capital letters denote differences among cultivars under control conditions, while lowercase letters denote significant differences under saline conditions (*p* < 0.05). Error bars represent standard errors.

The leaf Na data for peppers showed significant differences among different cultivars under control conditions. ‘Orange Habanero’ plants (138 mmol kg^−1^) had the highest leaf-Na concentration among all peppers under control conditions, with all the others averaging 12.5 mmol kg^−1^. Under salinity, ‘Orange Habanero’ (558 mmol kg^−1^) had significantly greater Na than all others (Fig. 4a). ‘Thai Hot’ accumulated the second-highest Na concentration (22 mmol kg^−1^) in leaves under salinity—although still over 25 times lower than ‘Orange Habanero’; yet, it had the greatest relative yield. ‘Ajvarski’ (329 mmol kg^−1^) accumulated the most Na in the roots under salinity (Fig. 4b).

Under control conditions, ‘Orange Habanero’ (241 mmol kg^−1^) accumulated the most Cl, significantly greater than all other cultivars, which averaged 131 mmol kg^−1^ (Fig. 4c). Under saline conditions, leaf Cl increased greatly in all cultivars with ‘Orange Habanero’ plants (915 mmol kg^−1^), ‘Ancho’ (841 mmol kg^−1^), and ‘Corbaci’ (791 mmol kg^−1^) having the highest Cl leaf accumulation (although not significantly from ‘Jimmy Nardello’ and ‘Costa Rican’), and ‘Thai Hot’ (501 mmol kg^−1^) had the lowest Cl accumulation. ‘Corbaci’ (344 mmol kg^−1^) accumulated significantly more Cl in the roots than ‘Costa Rican’ (212 mmol kg^−1^) and ‘Orange Habanero’ (238 mmol kg^−1^) in the saline treatment (Fig. 4d).

‘California Wonder’ (1,053 mmol kg^−1^) accumulated the most K in the leaves under control conditions, followed by ‘Ajvarski’ (995 mmol kg^−1^), ‘Corbaci’ (990 mmol kg^−1^), ‘Jimmy Nardello’ (977 mmol kg^−1^), and ‘Costa Rican’ (914 mmol kg^−1^), while ‘Orange Habanero’ plants (639 mmol kg^−1^) accumulated the least K (Fig. 4e). Under saline conditions, similar rankings occurred, except that ‘Jimmy Nardello’ (1000 mmol kg^−1^) had the highest K content, although not significantly higher than ‘Ajvarski’, ‘Costa Rican’, ‘California Wonder’ and ‘Corbaci’ (Fig. 4e). In roots, under salinity, there was no significant difference among cultivars in K concentrations (Fig. 4f). Two salt-tolerant cultivars, ‘Thai Hot’ and ‘Ancho’, maintained high K-salinity/K-control ratios of 1.10 each, and two salt-sensitive cultivars, ‘Corbaci’ and ‘Bloody Butcher’, had K-salinity/K-control ratios of 0.91 and 0.87, respectively (K data in Fig. 4e).

‘Corbaci’ accumulated significantly more Ca and Mg in the leaves than ‘Costa Rican’ and ‘Orange Habanero’ plants under both control and saline conditions (Supp Fig. S1e and S2e). ‘Thai Hot’ (552 mmol kg^−1^) accumulated significantly more Ca in the roots than all other cultivars under control conditions and significantly more Ca than ‘Jimmy Nardello’ (169 mmol kg^−1^) under salinity (Supp Fig. S1f). ‘Corbaci’ (181 mmol kg^−1^) accumulated the greatest amount of Mg in roots under the control condition, followed by ‘Jimmy Nardello’ (157 mmol kg^−1^) and ‘California Wonder’(145 mmol kg^−1^), while under the saline treatment, ‘California Wonder’ (128 mmol kg^−1^) had the greatest Mg concentration, significantly more than ‘Costa Rican’(84 mmol kg^−1^) (Supp. Fig. S2f).

‘California Wonder’ (5%) had significantly greater N in leaves under the control treatment. However, there was no significant difference in leaf-N accumulation in the cultivars under salinity (Supp. Fig. S3c).

### Effect of salinity on gene expression

For expression analyses, one salt-tolerant and one salt-sensitive genotype were selected each for eggplant, tomato, and pepper, based on their cumulative yield, relative yield, and ion uptake. The selected genotypes were ‘Long Purple’ (tolerant) and ‘Kazakhstan’ (sensitive) for eggplant, ‘Red Pear’ (tolerant) and ‘Bloody Butcher’ (sensitive) for tomato, and ‘Ancho’ (tolerant) and ‘Corbaci’ (sensitive) for pepper. A set of 12 genes known to be involved in salinity stress in model plants was used to study gene expression changes under control and salinity in three Solanaceae crops (Supplementary Table S1). The selected genes included Na^+^ transporters (*AKT1, AVP1, NHX1, NHX2, SOS1, SOS2*, and *SOS3*) and Cl^−^ transporters (*ALMT9, CCC, CLCc, CLCg*, and *SLAH3*).

The expression analyses showed that most genes were upregulated in roots under salinity compared to control for tomato and pepper (Fig. 6). However, eggplant roots did not show general upregulation under salinity. The overall expression of tested genes in pepper and eggplant was relatively low in leaves compared to roots (Fig. 6). This pattern was not consistent in tomato.

Of the genes involved in Na^+^ transport, *AKT1*, *SOS2*, and *SOS3* were significantly upregulated in roots under salinity compared to control in ‘Long Purple’, a relatively salt-tolerant cultivar of eggplant (Fig. 6). None of the Na^+^ transporter genes were differentially expressed in the roots of the salt-sensitive cultivar ‘Kazakhstan’. In leaves of ‘Long Purple’ (salt-tolerant cultivar), *AKT1* was upregulated under salinity compared to control, whereas *SOS1*, *SOS2*, and *SOS3* were upregulated under salinity compared to control in ‘Kazakhstan’ (Fig. 6).

In tomato, *AVP1*, *NHX1*, *NHX2* were upregulated in the roots of ‘Red Pear’ (salt-tolerant) under salinity (Fig. 6). Interestingly, *AKT1*, *AVP1*, *SOS1*, *SOS2*, and *SOS3* were upregulated in roots of the salt-sensitive ‘Bloody Butcher’ under salinity.

In pepper, three Na^+^ transporter genes (*AKT1*, *SOS1*, *SOS3*) were upregulated in roots of both ‘Ancho’ (salt-tolerant) and ‘Corbaci’ (salt-sensitive) under salinity (Fig. 6). *SOS2*, however, was upregulated in ‘Ancho’ roots but downregulated in ‘Corbaci’. In leaves of ‘Corbaci’, *AKT1* and *NHX1* were downregulated under salinity compared to control. On the other hand, for ‘Ancho’, none of the Na^+^ transporter genes were differentially expressed in leaves under salinity or control (Fig. 6).

The Cl^−^ transporter genes, *CLCc* and *CLCg* were upregulated, and *ALMT9* was downregulated under salinity compared to control in roots of salt-tolerant ‘Long Purple’ cultivar of eggplant (Fig. 6). *CLCg* and *SLAH3* were downregulated under salinity compared to control in leaves of ‘Long Purple’. None of the Cl^−^ transporters genes were differentially expressed in roots or leaves of ‘Kazakhstan’ (Fig. 6).

In tomato, *CCC*, *CLCc*, and *CLCg* were upregulated in roots of both ‘Red Pear’ and ‘Blood Butcher’ under salinity compared to control (Fig. 6). However, *ALMT9* and *SLAH3* were upregulated only in ‘Red Pear’ roots. Three Cl^−^ transporter genes, *ALMT9*, *CCC*, and *CLCc* were significantly downregulated in leaves of the salt-sensitive ‘Blood Butcher’ under salinity compared to control (Fig. 6).

In pepper, *CLCg* and *SLAH3* were upregulated in roots of both ‘Ancho’ (salt-tolerant) and ‘Corbaci’ (salt-sensitive) (Fig. 6). In addition, *ALMT9* and *CLCg* were upregulated only in ‘Corbaci’ roots. In ‘Corbaci’ leaves, *ALMT9* was upregulated, whereas *CLCg* was downregulated under salinity compared to control (Fig. 6). *SLAH3* was upregulated in both leaves and roots of ‘Ancho’ and ‘Corbaci’.

In the roots of salt-tolerant genotypes, there was a greater expression of some genes under control than for salt-sensitive genotypes for all three species. For instance, in eggplant, *SOS1*, *SOS2*, *ALTM9*, and *CCC* genes had at least twofold greater expression in ‘Long Purple’ roots under control than in ‘Kazakhstan’ under control (Fig. 6). Similarly, for tomato, *SOS1*, *SOS2*, and *CLCc* had at least double the expression levels in ‘Red Pear’ roots compared to ‘Bloody Butcher’ under control. For pepper, ‘Ancho’ roots showed more than two-fold expressions for *NHX1* and *CLCc* compared to ‘Corbaci’ under control (Fig. 6).

## Discussion

This study attempted to identify and characterize salinity tolerance mechanisms in three Solanaceae crops: eggplant, tomato, and pepper. We used eight, seven, and eight different heirloom cultivars for eggplant, tomato, and pepper, respectively, and evaluated their salinity tolerance by irrigating the plants with control (EC_iw_ = 0.65 dS m^−1^) or saline water (EC_iw_ = 4.0 dS m^−1^).

All species showed a significant reduction in fruit yield under salinity compared to control. The ratio of fruit yield under salinity divided by that under control is defined as the Salinity Tolerance Index (STI) and ranged from 0.52 to 1.14, 0.56 to 0.84, and 0.52 to 0.99 for eggplant, tomato, and pepper, respectively (Fig. 1). When averaged over all the cultivars tested in each species, eggplant showed the highest STI (0.86), followed by tomato (0.69), and then pepper (0.67) (Fig. 1). Thus, eggplant had better tolerance to salinity than tomato and pepper. Our data demonstrated large variability in cultivar salt tolerance and a difference in relative ranking as compared to the ranking in a widely cited review of crop salt tolerance where at EC_e_ = 4 dS m^−1^ the STI values were 0.80, 0.85, and 0.75 for eggplant, tomato, and pepper, respectively^6^. However, in this review crop salt tolerance was based on only 1–2 studies of each species, where salinity was well characterized and multiple salinity levels were tested. In addition, the greater eggplant salt tolerance that we report can be explained by the association of eggplant cultivars with their putative wild ancestor *Solanum insanum*, which showed higher tolerance to salinity than the domesticated *Solanum melongena* when both species were submitted to salinities ranging from 0 to 300 mM NaCl for 25 days^37^. This illustrates that vegetables have been primarily selected for yield and appearance and not for tolerance to abiotic stresses such as salinity, but such genes can still be found in wild cultivars and maybe heirloom cultivars.

The two top salt-tolerant eggplant cultivars were ‘Long Purple’ and ‘Turkish Orange’, and the two most salt-sensitive cultivars were ‘Black Beauty’ and ‘Kazakhstan’ (Fig. 1b). For tomato, ‘Red Pear’ and ‘Yellow Pear’ outperformed other cultivars in relative yield, and ‘Jaune Flamme’ and ‘Bloody Butcher’ were the worst performers (Fig. 1d). ‘Thai Hot’ and ‘Ancho’ cultivars of pepper were the most salt-tolerant, while ‘Corbaci’ and ‘Orange Habanero’ were the most salt-sensitive (Fig. 1f).

Previous research suggests that the ability of a plant to manage Na, Cl, and K during salinity stress is critical for its ability to tolerate salinity^22,23,38^. After evaluating these cultivars under the hot field climatic conditions of southern California for their whole crop cycle, it is reasonable to assume that, for the cultivars reported here as salt-sensitive, salt effects were related to ion toxicity rather than osmotic stress. Also, our relatively low salt stress (EC_iw_ = 4 dS m^−1^), corresponds to roughly—0.16 MPa of osmotic pressure, too low to create water stress. In our experiment, all three Solanaceae crops had high Na concentrations in roots compared to leaves (Figs. 2a,b, 3a,b and 4a,b), with the notable exception of ‘Orange Habanero’ plants, suggesting that there is a distinct mechanism regulating translocation of Na from roots to shoots. Total leaf concentrations of Na were drastically different in all three Solanaceae crops tested. Except for ‘Orange Habanero’, pepper and eggplant had less than tenfold leaf Na accumulation compared to tomato (Figs. 2a, 3a, and 4a). These observations indicate that eggplant and pepper plants have a more efficient mechanism to regulate the translocation of Na from roots to shoots.

Considering the market value of hot peppers for their international culinary appreciation and the pharmacological and commercial value of capsaicin and dihydrocapsaicin, ‘Thai Hot’ (*C. annuum* L.) and ‘Orange Habanero’ pepper (*C. chinense* Jacq.) were included as representatives of hot peppers. ‘Thai Hot’ was the most salt-tolerant of all the pepper species having no decrease in fruit yield or size under salinity (Fig. 1e), whereas ‘Orange Habanero’ was one of the most salt-sensitive pepper cultivars (Fig. 1f). ‘Orange Habanero’ has a medium size for a hot pepper and is much larger than ‘Thai Hot’, but its hollow fruit and its thin flesh compared to other peppers (e.g., ‘Ancho’) account for its low fruit yield, even under control conditions. ‘Orange Habanero’ is a very aromatic pepper favored by its mild heat^39^, attractive orange color, aroma when ripe, and long shelf life, making it one of the most commonly found hot peppers in supermarkets and fresh market stands.

Among peppers, only ‘Orange Habanero’ showed a high accumulation of Na in leaves (527.8 mmol kg^−1^) under salinity; all the other cultivars accumulated between 9.8 and 22.4 mmol kg^−1^ (Fig. 4a). ‘Orange Habanero’ had more than 23-fold difference in leaf Na accumulation compared to the rest of the pepper cultivars, indicating that the specific difference in Na translocation from roots to shoots of this species (*Capsicum chinense*) may reflect the possible center of origin of ‘Orange Habanero’ compared to the other pepper plants belonging to the species *C. annuum*. ‘Orange Habanero’ ancestors seem to have originated from tropical climates from Central and South America, such as Brazil, Peru, and Surinam, and the Pacific Islands, such as Hawaii, Fiji, Samoa, Guam, etc.^40,41^ and later on spread throughout the New World into places of similar climates, such as Florida, St. Augustine, and Puerto Rico^41^. This striking difference in the control of Na^+^ transport from roots to shoots is typical of glycophyte plants that originated and evolved in areas where Na^+^ was not a selective pressure in the evolution of the species. This hypothesis is consistent with the fact that soils of the Amazonian basin have very low available Na and Ca^42^. On the other hand, the greatest diversity of cultivated forms of *C. annuum* were reported to have originated mainly from Central Mexico with a secondary center in Guatemala^41^.

For several plant species, cultivars that efficiently exclude Na are the most salt-tolerant^30^. Our results showed that tomato and pepper followed these anticipations; however, eggplant data did not follow the expected trend. ‘Bloody Butcher’ and ‘Jaune Flamme’, two tomato cultivars with the lowest STI, accumulated the highest leaf Na under the salinity treatment (Fig. 3a). Conversely, ‘Red Pear’, the cultivar with the highest STI, accumulated the least amount of Na in the leaves under salinity. On the other hand, ‘Red Pear’ had a relatively high root Na concentration (Fig. 3b). The low Na leaf concentration of ‘Red Pear’ is thus likely related to its ability to restrict Na translocation from root to shoot rather than restrict Na uptake. For pepper, ‘Orange Habanero’, with the second lowest STI, had the greatest leaf accumulation of Na by far (Fig. 4a). In addition, ‘Orange Habanero’ had one of the lowest root Na concentrations, thus demonstrating its inability to restrict Na translocation from root to shoot (Fig. 4b) that again may be related to its evolution in tropical countries with soils poor in Na^+^. ‘Corbaci’, which had the lowest STI, was the third largest in leaf Na accumulation (Fig. 4a). ‘Ancho’, one of the best performing cultivars, had the lowest leaf Na concentration and has been recently reported, along with other *C. annuum* species, as one of the landraces that may have its center of origin in central to northern Mexico^43^, a region of relatively high soil salinity. Although leaf Na concentration showed association with salt tolerance of different cultivars, it was not always perfect. For instance, ‘Thai Hot’, a cultivar with the highest STI, accumulated a high amount of Na in leaves (Figs. 1f and 4a). These observations suggest that different cultivars may vary in component traits of the salt tolerance mechanisms, emphasizing the complexity of the plant responses to salinity stress.

Similar to leaf Na concentration data, leaf Cl data showed some association with STI for tomato and pepper, but not for eggplant. In tomato, ‘Red Pear’ and ‘Yellow Pear’, two cultivars with high STI, had low leaf Cl concentrations (Fig. 3c). On the other hand, salt-sensitive cultivars, ‘Jaune Flamme’ and ‘Bloody Butcher’, had higher leaf Cl concentrations (Fig. 3c). For pepper, ‘Corbaci’ was the most salt-sensitive, had the highest leaf Cl concentration. In contrast, ‘Thai Hot’ that was the most salt-tolerant cultivar, had the lowest leaf Cl concentration (Fig. 3c). However, ‘Ancho’, one of the most salt-tolerant cultivars, also accumulated a high amount of Cl in leaves. These observations suggest that although leaf Cl concentration is an important parameter during salinity stress, the overall salinity tolerance of a cultivar is regulated by a complex of several factors.

Potassium is an important macronutrient in plants that is involved in several physiological and biochemical processes, including cell osmoticum, pH regulation, stomatal opening/closing, regulation of membrane potential, and enzyme activity^44^. As Na^+^ competes with K^+^ for binding sites, high salt negatively affects the K content in plants^45^. Most plants show decreased leaf K concentration under salinity^38,46^; this includes tomato^15,47–49^, where yield loss in NaCl solution was attributed to Na-induced K deficiency^50^. However, in tomato studies with multiple salinity levels, leaf K levels did not significantly decrease until large yield losses occurred^11,22^. In another tomato study with two species, the *L. cheesmanii* species from the Galapagos Islands showed less reduction in vegetative growth (more salt-tolerant ) than did *L. esculentum* Mill cv. VF 36, but the decrease in leaf K with salinity was more marked for the more salt-tolerant species *L. cheesmanii*^51^.

In our study, pepper cultivars showed no change in leaf K (Fig. 4e), but tomato cultivars showed an average of 21% decrease in leaf K concentration under salinity compared to control (Fig. 3e). Interestingly, eggplant cultivars showed an average of 19% increase in leaf K concentration under salinity than control (Fig. 3e). Tomato plants are considered to be deficient in K when mature leaf K concentrations are below 380 mmol per kg^52^. Increasing K application in tomato from 200 to 400 mg L^−1^ under saline conditions (35 mM NaCl) resulted in increased leaf and root K and decreased leaf and root Na^53^. However, fruit yield was unaffected by K addition. With this criterion applied to other Solanaceae species, none of our eggplant or pepper cultivars were deficient in K (Figs. 2e and 4e). However, under salt stress, ‘Jaune Flamme’ tomato plants were deficient in K (300 mmol kg^−1^), and ‘Garden Delight’ and ‘Yellow Pear’ were borderline deficient (Fig. 3e). As ‘Jaune Flamme ‘had the most significant yield loss of all tomato cultivars (Fig. 1), it is reasonable to attribute this loss to K deficiency under salt treatment. Tomato shoot dry weight reduction of 20% was reported when leaf K was 250 mmol per kg^52^.

Previous studies with pepper also show decreasing K with increasing salinity for whole plant^54^ as well as leaf K concentration^21^. Previous studies with eggplant generally showed decreasing K with increasing salinity^55^. However, a hydroponic study with 4 eggplant cultivars showed no significant changes in leaf K at 20 and 40 mM NaCl treatments while vegetative fresh weight significantly decreased^56^. At the highest salt levels, the more salt-sensitive cultivar (20% relative biomass) experienced a greater decline in leaf K than the more salt-tolerant one (65% relative biomass)^56^.

As discussed above, eggplant showed relatively little Na accumulation in leaves as compared to tomato (Figs. 2a, 3a, and 4a). It is known that the exclusion of Na^+^ from plant tissue leads to high K^+^ levels^57^. Hence, the differences in relative leaf K concentration under salinity compared to control (K-salinity/K-control) among three Solanaceae crops may result from differences in exclusion of Na^+^ from leaves.

**Figure 5:**
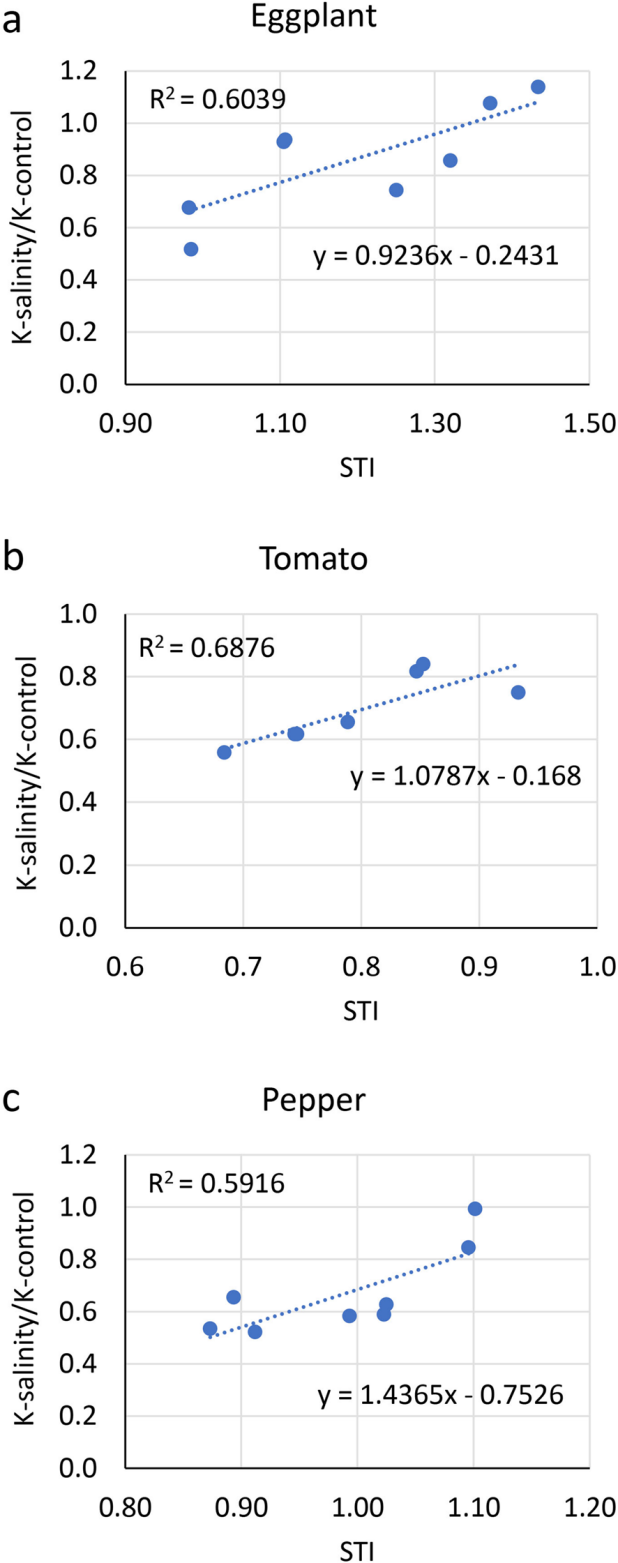
Relationship of the ratio of leaf K under salinity and control (K-salinity/K-control) with salt tolerance index (STI) in eggplant, tomato, and pepper. (**a**) Eggplant. (**b**) Tomato. (**c**) Pepper. Dots represent the mean values of different cultivars.

We observed a strong association between salt tolerance and leaf K-salinity/K-control ratio for all Solanaceae species (Fig. 5). Cultivars with high STI also had a high K-salinity/K-control ratio in leaves. STI and K-salinity/K-control ratio depicted high correlations with R^2^ values of 0.60, 0.69, and 0.59 for eggplant, tomato, and pepper, respectively (Fig. 5). Net leaf K concentration did not show an association with salinity tolerance. For instance, pepper cultivar ‘Thai Hot’ (most salt-tolerant) had a lower leaf K concentration under salinity compared to ‘Corbaci’ (most salt-sensitive) (Fig. 4e). Hence, our data suggest that rather than net K concentration in leaf, the K-salinity/K-control ratio is more critical in determining salinity tolerance of a genotype in Solanaceae. In a previous study, the main feature of the salt-tolerant cultivar of eggplant was shown to be its ability to absorb K^+^ by upregulation of several K transporters^32^. In an almond study focusing on characterizing salinity responses in 14 almond rootstocks, the percent reduction in leaf-K concentration under salinity was highly correlated with salinity tolerance^38^.

**Figure 6:**
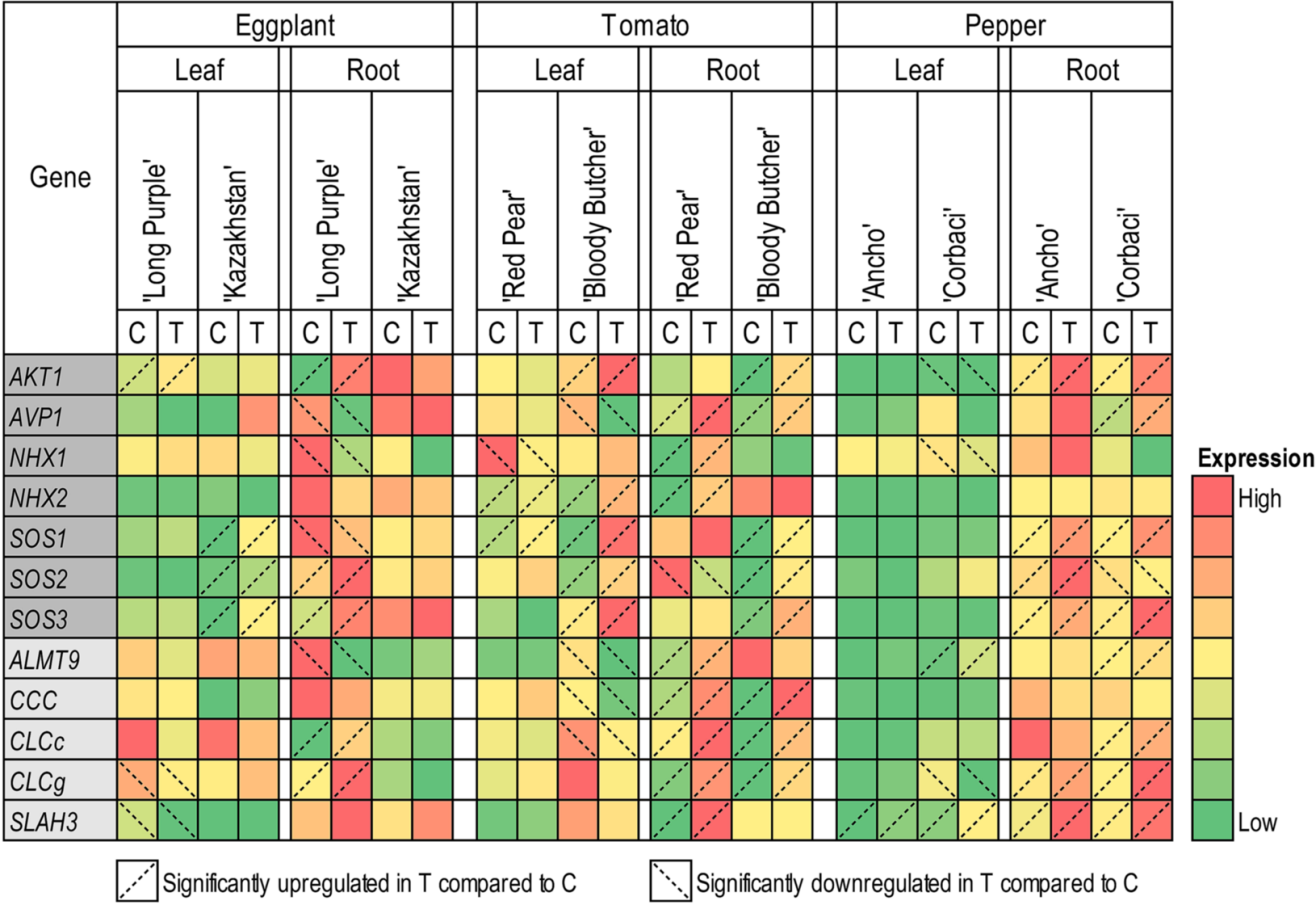
Heatmap representing the relative expression of salt stress-related genes in roots and leaves of a salt-tolerant and a salt-sensitive cultivar each of the three Solanaceae crops, eggplant, tomato, and pepper. Expression values for each gene are color-coded to depict the fold-change in different cultivars. The genes highlighted in dark gray represent genes involved in Na transport, and the genes highlighted in light green represent genes involved in Cl transport. C, control; T, salinity treatment.

To understand the roles of different players involved in salinity stress, expression analyses were conducted for genes involved in Na^+^ and Cl^−^ transport using a salt-tolerant and a salt-sensitive cultivar for each of the three Solanaceae crops. The selected salt-tolerant and salt-sensitive cultivars include ‘Long Purple’ and ‘Kazakhstan’, ‘Red Pear’ and ‘Bloody Butcher’, and ‘Ancho’ and ‘Corbaci’, for eggplant, tomato, and pepper, respectively. The general expression levels of several genes were higher in roots compared to leaves in eggplant and pepper (Fig. 6). Given that most of the genes used in the study regulate Na^+^ and Cl^−^ transport, and that roots play an important role in the uptake and translocation of these ions, higher expression levels of these genes in roots would be expected. The low expression levels of genes involved in Na^+^ transport in leaves of eggplant and pepper may also explain why Na concentration was more than tenfold lower in leaves of eggplant and pepper than tomato (Figs. 2a, 3a, and 4a).

It has been observed in some plant species that the basic expression levels of several genes involved in salinity stress are higher in salt-tolerant genotypes compared to salt-sensitive genotypes^38^. Based on the genes tested in this investigation, we did not see that general trend (Fig. 6). However, *CCC* and *SOS2* showed at least twofold expression in the roots of ‘Long Purple’ compared to ‘Kazakhstan’ eggplants, both under control and salinity, which may indicate that the basic expression levels of some genes may be critical in imparting salinity tolerance in salt-tolerant genotypes compared to the salt-sensitive ones (Fig. 6).

Several genes showed differential expression under salinity compared to control in Solanaceae species. In eggplant, three genes involved in Na^+^ transport, *AKT1*, *SOS2*, and *SOS3,* were significantly induced under salinity in the roots of salt-tolerant cultivar Long Purple, while none of the genes involved in Na transport were induced in the salt-sensitive cultivar ‘Kazakhstan’ (Fig. 6). These observations demonstrate that ‘Long Purple’ is better equipped to tolerate salinity by excluding Na^+^ from roots. Two genes involved in Cl^−^ transport, *CLCc,* and *CLCg,* were induced under salinity compared to control in roots of ‘Long Purple’ but not in ‘Kazakhstan’, which may explain greater Cl concentration in ‘Kazakhstan’ leaves as compared to ‘Long Purple’.

In tomato, three genes involved in sequestering Na^+^ in the vacuole, *NHX1*, *NHX2,* were upregulated under salinity in roots of ‘Red Pear’ but not in ‘Bloody Butcher’, suggesting that ‘Red Pear’ may better partition excess Na^+^ in the vacuole, protecting the cytoplasm from Na^+^ toxicity (Fig. 6). ‘Red Pear’s root Na concentration was higher than ‘Bloody Butcher’, but due to effective compartmentalization of Na^+^ in the vacuole, the relative fruit yield performance of ‘Red Pear’ was better under salinity, making it salt-tolerant. Several important genes involved in Na^+^ uptake *SOS1*, *SOS2*, and *SOS3* were upregulated in salt-sensitive tomato cultivar ‘Bloody Butcher’ (Fig. 6), suggesting that the SOS pathway is active in ‘Bloody Butcher’, which is justified by its second-lowest root Na concentration among all tomato cultivars (Fig. 3b). However, ‘Bloody Butcher’ showed significant downregulation of three Cl^−^ transporter genes, *ALMT9*, *CCC*, and *CLCc,* in leaves (Fig. 6), which may have contributed toward its salt-sensitivity compared to ‘Red Pear’.

In pepper, most of the genes were upregulated both in roots of salt-tolerant (‘Ancho’) and salt-sensitive (‘Corbaci’) cultivars. However, *SOS2* was upregulated in the roots of ‘Ancho’, but it was downregulated in the roots of ‘Corbaci’ (Fig. 6), which may be the reason for the better performance of ‘Ancho’ as compared to ‘Corbaci’ under salinity.

## Conclusion

Our selection of cultivars of heirloom vegetables based on different geographic regions produced expected, as well as less expected, results. In general, based on the salt tolerance index (STI), eggplant was the most salt-tolerant species (STI = 0.86), followed by tomatoes (STI = 0.69), and then peppers (STI = 0.67), the most sensitive among the three crops.

Eggplant, a crop that originated in Asia and cultivated for centuries in Asia, Africa, Europe, and the Near East, has controversial accounts for its center of origin but is mostly cultivated in China, India, Egypt, Turkey, and Japan^58^. The cultivars ‘Long Purple’ (from India) and ‘Turkish Orange’ (from Turkey) demonstrated the highest tolerance to salinity as might be expected, but ‘Ping Tung’ (from Taiwan) and ‘Bangladeshi Long’ (from India) were close contenders and lost minimal fruit yield under salinity making eggplant, in general, the most salt-tolerant of the three Solanaceae crops. These findings suggest that salt-tolerant eggplant cultivars could potentially be used as rootstocks for more salt-sensitive tomato cultivars. Besides salinity tolerance, some eggplant cultivars also offer other benefits over tomato rootstocks, such as resistance to different bacterial wilts (*Ralstonia solanacearum* or *Pseudomonas solanacearum*), which can cause up to 100% economic losses in tomato crops^59^. It also should be noted that although ‘Black Beauty’ had the lowest relative yield under the salinity tested (EC_iw_ = 4.0 dS m^−1^), it still had the significantly greatest fruit yield among eggplant cultivars under salinity, suggesting that it still could be a feasible cultivar to be used under moderate irrigation water salinities.

Tomatoes are well adapted to a variety of climates and are widely distributed in habitats ranging from the arid Pacific coast at sea level to altitudes above 3000 m above sea level in the Andes with its wild species reported as native to Bolivia, Peru, Ecuador, Chile, Colombia, and Galapagos Islands ^60^. Despite its wide genetic variability, little progress has been made towards establishing tomato cultivars that are tolerant to salinity. In our study, the most salt-tolerant tomato cultivars were ‘Red Pear’ and ‘Yellow Pear’. These results were surprising as we expected other cultivars, especially ‘Thessalonniki’ (from Greece) and possibly ‘Jaune Flamme’ (France), to be most salt-tolerant as both are from Mediterranean, semi-arid climates. However, these two were the most salt-sensitive cultivars.

As might be expected, ‘Orange Habanero’ (*Capsicum chinense* Jacq.), which originated from low-salinity Amazonian regions, had the weakest mechanism of controlling Na transport from roots to shoots. At the same time, all other cultivars belonging to *C. annuum*, and believed to have their center of origin in semi-arid Mexico, had similar capabilities to restrict Na to their roots and prevent its translocation to the shoots. Crossing (or grafting) *Capsicum chinense* with (or onto) a more salt-tolerant *C. annuum* cultivar (e.g., ‘Thai Hot’ or ‘Ancho’) could potentially make ‘Orange Habanero’ more tolerant to salinity.

Response to salinity differed significantly among species and cultivars. The most sensitive tomato ‘Jaune Flamme’, exhibited leaf K deficiency under the salt treatment. The most salt-tolerant pepper cultivars of the species *Capsicum annum* were ‘Thai Hot’ with high leaf Na and low leaf Cl and ‘Ancho’, with relatively high root K. The most sensitive cultivar, ‘Corbaci’, was characterized by high leaf Ca and Mg and high root Cl.

Expression analysis revealed that salinity treatment-specific induction of genes was more important than genotype-specific expression in Solanaceae. The differences in relative yield were not explainable by one single mechanism, but rather by multi-component traits that were critical during salinity stress. The primary component traits included the ability to exclude Na, restrict Na transport to leaves, sequester Na^+^ and Cl^−^ in vacuoles, and accumulate K.

Evaluation of heirloom cultivars of diverse geographic origins under field conditions in the dry and hot semiarid climate of southern California provided us with a unique opportunity to genetically characterize these genotypes for their salinity tolerance. Understanding the genetic networks involved in salinity tolerance in Solanaceae will provide additional tools for breeders to develop varieties for regions affected by high salinity.

## Materials and methods

### Plant material

A field experiment was conducted at the U.S. Salinity Laboratory in Riverside, California, from March 2018 to October 2018 to evaluate the salt tolerance of 8 different eggplant cultivars, 8 different tomato, and 8 different peppers cultivars. Most of the plants of ‘Cherokee Purple’ cultivar of tomato died due to disease; hence this cultivar was eliminated and only 7 tomato cultivars were used for the analyses. Heirloom cultivars of eggplant (*Solanum melongena* L.) that were tested were: ‘Bangladeshi Long’, ‘Black Beauty’, ‘Kazakhstan’, ‘Listada de Gandia’, ‘Long Purple’, ‘Ping Tung’, ‘Tadifi’, and ‘Turkish Orange’. The peppers tested were (mostly *Capsicum annuum* L.): ‘Ajvarski’, ‘Ancho’ (Poblano), ‘California Wonder’, ‘Corbaci’, ‘Costa Rican’ (a sweet hybrid), ‘Orange Habanero’ pepper (*Capsicum chinense* L.), ‘Jimmy Nardello’, and ‘Thai Hot’. The tomato heirlooms that were tested were: ‘Bloody Butcher’, ‘Cherokee Purple’, ‘Garden Delight’, ‘Jaune Flamme’, ‘Quarter Century’, ‘Red Pear’, ‘Thessaloniki’, and ‘Yellow Pear’.

### Experimental design and treatments

The experiment consisted of 576 plants (192 plants of each species) arranged in a split-plot design with two salt levels in the main plots arranged in randomized complete blocks with three replications. Plants were transplanted in May 2018 with 32 plants per row and a total of 18 rows. Seedlings were established one month earlier in the greenhouse under non-saline conditions. The rows were irrigated with non-saline irrigation water (control was Riverside city tap water with EC_iw_ = 0.65 dS m^−1^) and saline water (EC_iw_ = 4.0 dS m^−1^). Each plant was irrigated using one pressure compensated 2 L h^−1^ micro-sprinkler. The saline water composition consisted of mixed salts giving a composition, of Ca = 10.9, Mg = 7.5, Na = 21.4, K = 1.0, sulfate = 12.9, Cl = 27.5 and NO_3_^−^ = 0.4, where concentrations are expressed in mmol_c_ L^−1^. Before planting, 500 g of dried steer manure was incorporated into the soil used to fill each of the holes augered into the field for accepting transplants.

### Irrigation management

The field plot was irrigated once to two times a day with frequency and time intervals depending on the water requirements. From June to October, each plant received between 1 and 2.26 L per day on non-raining days. When a rain event occurred, irrigation was adjusted accordingly. Each plant was fertilized with 10 g of Osmocote Plus (a slow-release fertilizer with N—15%, P—9%, K—12%, Mg—1.3%, S—6%, B—0.02%, Cu—0.05%, Fe—0.46%, Mn—0.06%, Mo—0.02%, Zn—0.05% by weight) in August of 2018. The local CMIS weather station data indicated that for the experimental period, the potential reference crop ET (ET_0_) was 8.31, 20.42, 18.67, 14.88, and 10.9 cm for June, July, August, September, and October, respectively. The corresponding total water applications were 13, 62.3, 70.1, 60.0, and 47.1, L/plant, respectively. We estimate a canopy cover of 0.5 and a crop coefficient factor of 1.5 to account for the water requirements of plants grown in a small plot environment. The estimated leaching fraction was thus calculated as an average of 0.37 over the experimental period. Using the relationship between irrigation water salinity and leaching fraction, the average rootzone salinity, expressed as EC saturation extract, was calculated as 3.7 dS m^−1^ for the saline treatment.

### Plant measurements

Fruit yields of each plant were collected weekly from July to October, from which we calculated the average fruit weight per plant. A composite sample of 9–30 fully expanded leaves for each cultivar and each treatment were collected in September 2018. Samples were weighed, washed, oven-dried, reweighed, microwave digested, and subsequently analyzed for Ca, Mg, Na, K, P, S, Fe, Cu, Mn, and Zn by Perkin Elmer Optima 3300 DV ICP OES (WinLab 32, 2010). Chloride concentrations were determined by wet digestion of the plant material and then analyzed by an amperometric chloride titrator. A composite sample of roots of each cultivar from each row was taken between October 30th to November 1st and analyzed as described above.

### Statistical analysis

The SAS/STAT® software package was used for three-way analyses of variance (ANOVA), followed by Tukey and Tukey–Kramer pair-wise comparison of means. Differences with an alpha = 0.05 or less were considered significant. ANOVA and Tukey’s test were used to analyze multiple variables using the general linear model proc GLM. We also used the CORR procedure to perform correlation analyses.

### Primer design for expression analyses

Genes involved in Na^+^ and Cl^−^ transport were identified using functional conservation with the genes identified in Arabidopsis^29^. Basic Local Alignment Search Tool (BLAST) analysis was utilized to identify corresponding genes in eggplant, tomato, and pepper. The gene with the highest homology was used to design primers for qRT-PCR analyses. To ensure no nonspecific amplification from contaminating DNA, at least one was designed from two exons flanking an intron.

### Expression analyses

Leaf and root tissue samples were harvested from one salt-tolerant and one salt-sensitive cultivar each of eggplant, tomato, and pepper (2 cultivars × 3 crops × 2 tissue types × 3 replications × 2 salinity levels), 48 h after initiation of salinity treatment. Young tissue samples were frozen immediately in liquid nitrogen, and RNA was isolated using TRIzol reagent (Invitrogen, Carlsbad, CA, USA). From isolated RNA, contaminating DNA was removed using DNase I, following the manufacturer’s instructions (Thermo Scientific, Waltham, MA, USA). The qRT-PCR experiment was conducted using iTaq Universal SYBR Green One-Step Kit in BioRad CFX96 system (Bio-Rad Laboratories, Hercules, CA, USA). For qRT-PCR reactions, 10 µL volume was used that contained 100 ng total RNA, 0.125 µL iScript Reverse Transcriptase, 0.75 µM of each of the primers, and 5 µl of 2 × one-step SYBR Green Reaction mix. The PCR reactions were carried out using the following program: 50 °C for 10 min, 95 °C for 1 min, then 40 cycles of 95 °C denaturation for 10 s, 57 °C annealing for 30 s, and 68 °C extension for 30 s. Four samples were used as inter-plate controls for normalization of expression in different plates. Cyclophilin (AF126551), 18S ribosomal RNA (X67238), and glyceraldehyde-3-phosphate dehydrogenase (U17005) were used as reference genes for eggplant. Actin (TC194780), protein phosphatase 2A catalytic subunit (AY325817), and ribosomal protein L2 (X64562) were used as reference genes for tomato. Actin (GQ339766), glyceraldehyde-3-phosphate dehydrogenase (AJ246009), and EF1α (AY496125) were used as reference genes for pepper. Relative expression of each gene was determined by comparing the cycle threshold of each gene to the reference genes, and differentially expressed genes were identified. To test the amplification specificity, melt curve analysis was used by ramping the temperature to 95 °C for 10 s, then back to 65 °C for 5 s, followed by incremental increases of 0.5 °C/cycle up to 95 °C.

The use of plants in the present study complies with international, national and/or institutional guidelines.

## Supplementary Information

Supplementary Information.

## Data Availability

All data supporting this study are included in the article and its supplementary material.

## Abbreviations

PCR: Polymerase chain reaction
qRT-PCR: Quantitative reverse transcription-polymerase chain reaction
ORAC: Oxygen radical absorbance capacity
BLAST: Basic local alignment search tool
