# Supplementary material for: Linking genetic determinants with salinity tolerance and ion relationships in eggplant, tomato and pepper
Source: Sci Rep. 2021 Aug 11;11:16298. doi: 10.1038/s41598-021-95506-5 (PMC8357798; doi:10.1038/s41598-021-95506-5)
Supplement: Supplementary file 1 — Supplementary Information. [file 41598_2021_95506_MOESM1_ESM.pdf]

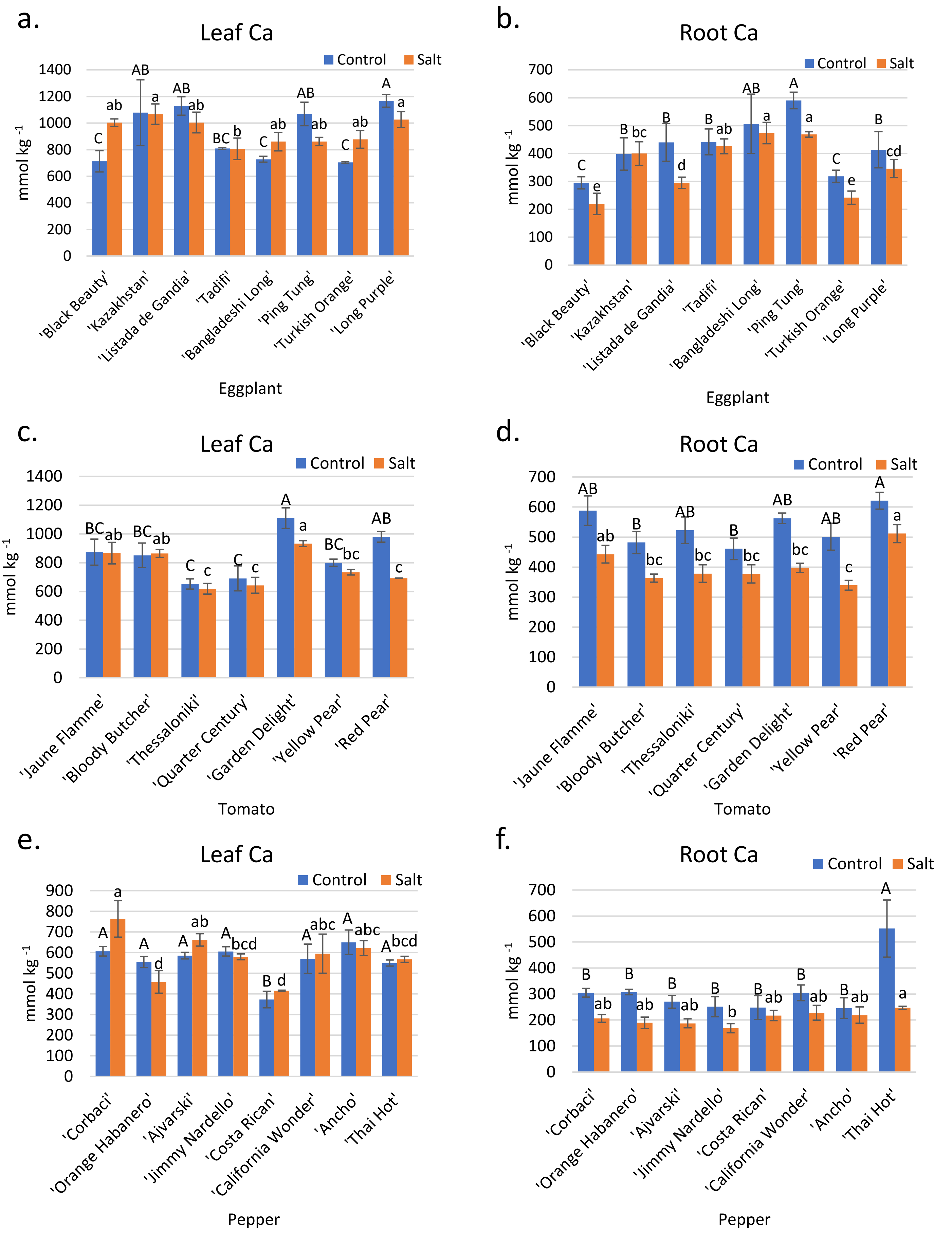

Supplementary Figure S1. Tissue Ca concentrations of different cultivars of eggplant, tomato, and pepper. a) Eggplant leaf Ca concentrations. b) Eggplant root Ca concentrations. c) Tomato Leaf Ca concentrations. d) Tomato root Ca concentrations. e) Pepper leaf Ca concentrations. f) Pepper root Ca concentrations. Capital letters denote significant differences among cultivars under control condition ($p<0.05$). Lowercase letters denote significant differences among cultivars under saline conditions ($p<0.05$). Error bars represent standard errors.

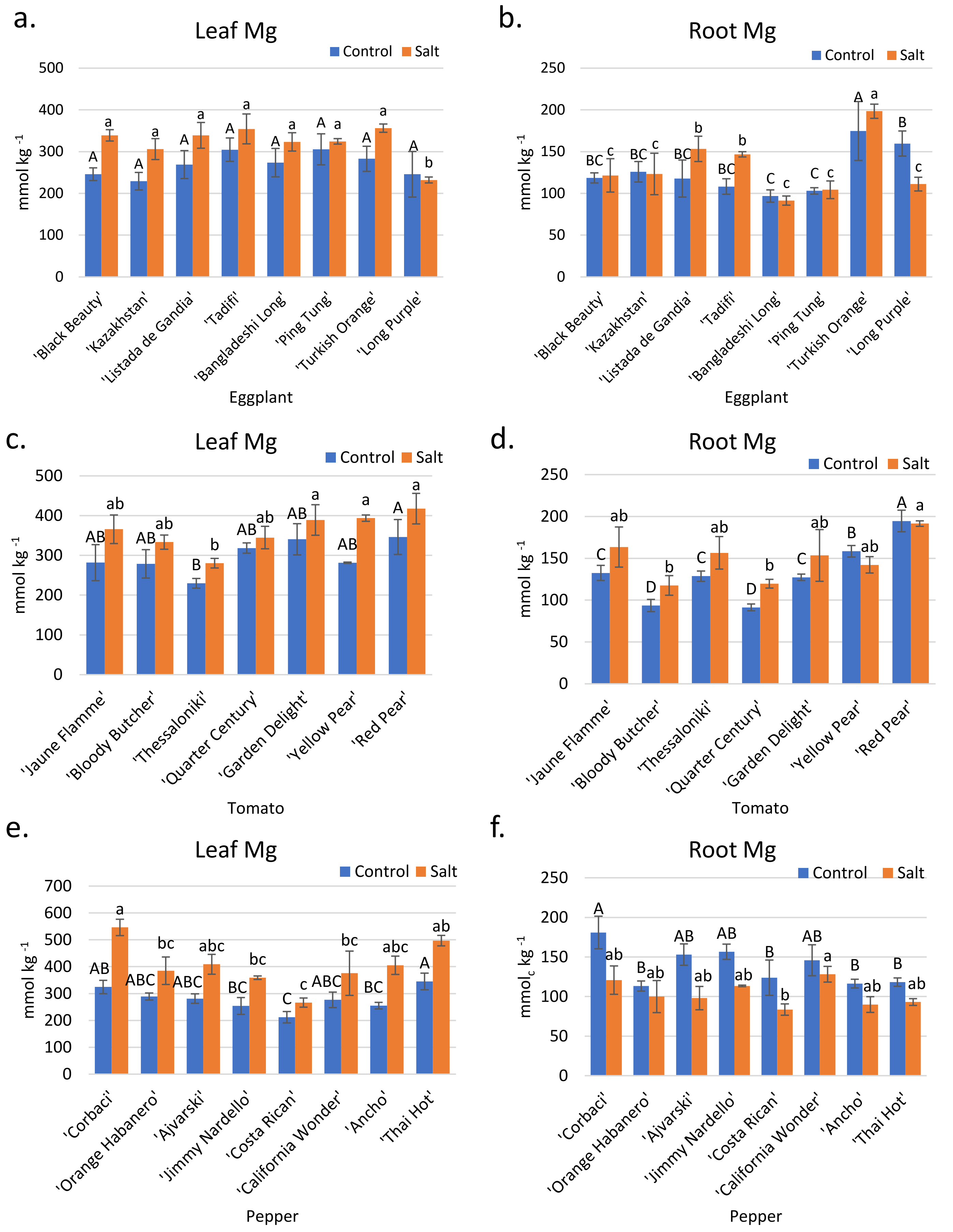

Supplementary Figure S2. Tissue Mg concentrations of different cultivars of eggplant, tomato, and pepper. a) Eggplant leaf Mg concentrations. b) Eggplant root Mg concentrations. c) Tomato Leaf Mg concentrations. d) Tomato root Mg concentrations. e) Pepper leaf Mg concentrations. f) Pepper root Mg concentrations. Capital letters denote significant differences among cultivars under control condition ($p<0.05$). Lowercase letters denote significant differences among cultivars under saline conditions ($p<0.05$). Error bars represent standard errors.

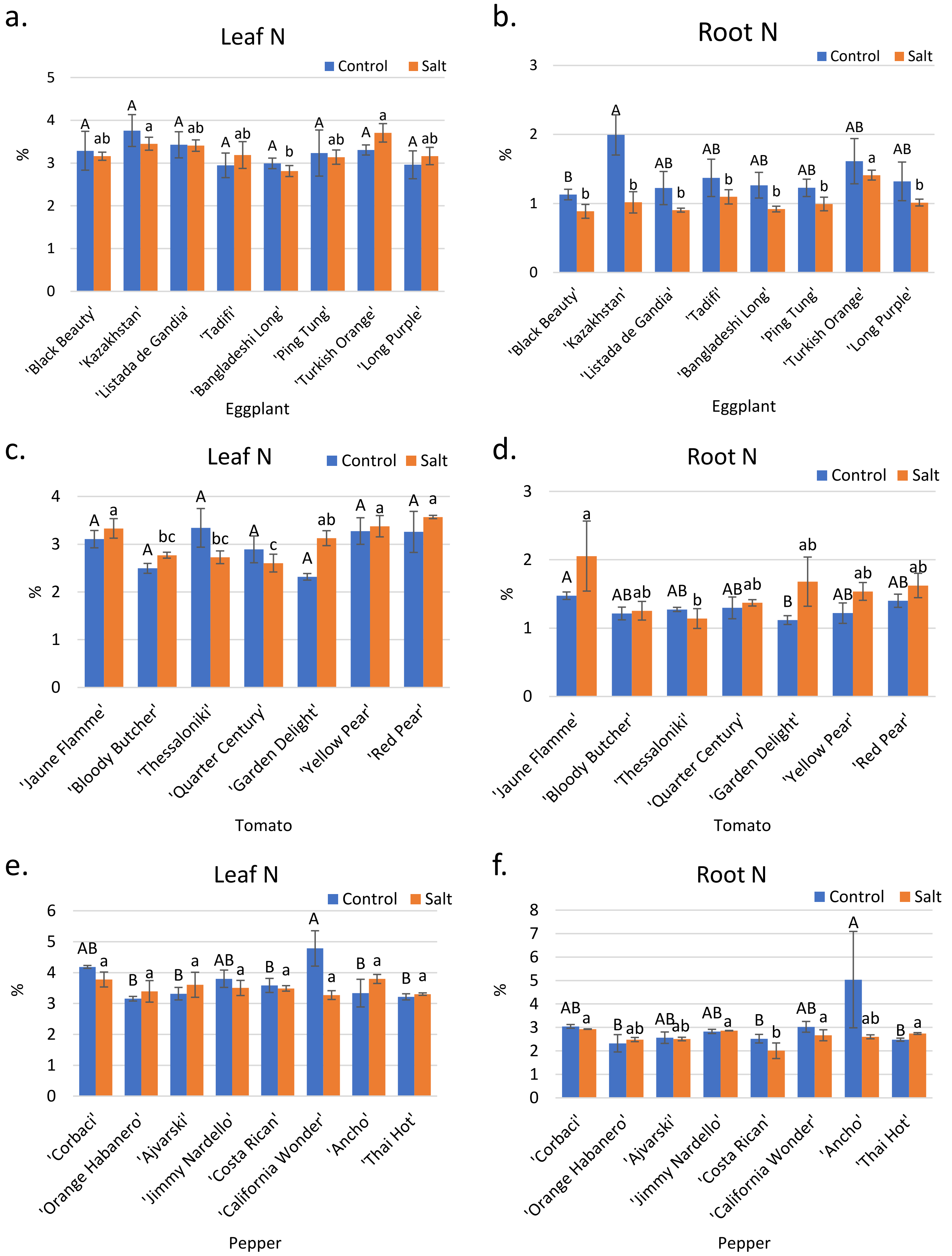

Supplementary Figure S3. Tissue N concentrations of different cultivars of eggplant, tomato, and pepper. a) Eggplant leaf N concentrations. b) Eggplant root N concentrations. c) Tomato Leaf N concentrations. d) Tomato root N concentrations. e) Pepper leaf N concentrations. f) Pepper root N concentrations. Capital letters denote significant differences among cultivars under control condition ($p<0.05$). Lowercase letters denote significant differences among cultivars under saline conditions ($p<0.05$). Error bars represent standard errors.

Supplementary Table S1. List of primers used for qRT-PCR analyses of eggplant, tomato, and pepper

Crop	Gene	Primer Name	Sequence (5' to 3')
Eggplant	*SmAKT1*	Sm_AKT1_F	TCCAGCTGGTCTCCGAG
		Sm_AKT1_R	ACTAGATCCACAGCTCCTGTTA
	SmAVP1	Sm_AVP1_F	GGAAAGGGATTTGCCATTGG
		Sm_AVP1_R	CTTGGGGGTCAAGACATCG
	SmNHX1	Sm_NHX1_F	ATTTTGGCAGGCACTCCA
		Sm_NHX1_R	CACTTAAATAGAATAGTTCAGCCAACA
	SmNHX2	Sm_NHX2_F	ATATCATTAGCTGCTATTGGCATTTT
		Sm_NHX2_R	TTGCTCCAATCGCTAGGTAATC
	SmSOS1	Sm_SOS1_F	AGCATGATAAGCAACACAAGAAG
		Sm_SOS1_R	CGATCTGGTCTAACTTCTGGAAA
	SmSOS2	Sm_SOS2_F	GGCTTCACGAGGTTTTAGCT
		Sm_SOS2_R	GATGAACAATTTTATCAAAAAGCTCTCC
	SmSOS3	Sm_SOS3_F	GACAGCTTTTACGGAGAGTGA
		Sm_SOS3_R	CTTCCTTGTGAATTAGTCCATCATC
	SmALMT9	Sm_ALMT9_F	CTAGAAGGTTGTATCAGTGAATATCTGA
		Sm_ALMT9_R	CTGCTGATCTGTAGCCATTGT
	SmCCC	Sm_CCC_F	GCCATGAAGGGTGGTGG
		Sm_CCC_R	ACATACATTGCACCACCAATTG
	SmCLCc	Sm_CLCc_F	GCTTCATGGTGGCGAAGT
		Sm_CLCc_R	CAGAATTGAATGCAAGATCTGAGT
	SmCLCg	Sm_CLCg_F	GAACGAGAGAAGAGCTGCTTAC
		Sm_CLCg_R	AGGATGGCAAGGTGCAAA
	SmSLAH3	Sm_SLAH3_F	GCTAAGGGCTTCTGAGGAAAG
		Sm_SLAH3_R	GCCAAGAATGATACCAAACGATG
	SmCYP	Sm_CYP_F	ATCCTGTCCATGGCTAATGC
		Sm_CYP_R	ATGCCCTCAACAACTTGTCC
	Sm18cRNA	Sm_18sRNA_F	TAGTTGGACTTTGGGATGGC
		Sm_18sRNA_R	AGAGCGTAGGCTTGCTTTGA
	SmGAPDH	Sm_GAPDH_F	CCGCTCCTAGCAAAGATGCC
		Sm_GAPDH_R	ACCCTCCACAATGCCAAACC
Tomato	*SlAKT1*	Sl_AKT1_F	CGCGTTGCATATCGCTG
		Sl_AKT1_R	GCCTTCTGAATCTCTACTATTGGG
	SlAVP1	Sl_AVP1_F	AAGAATTGGGAGCTCTTTTTATGTG
		Sl_AVP1_R	TTGGACAGGGCTGTAAGC
	SlNHX1	Sl_NHX1_F	ATGCAGGGTTCCAGGTAAAA
		Sl_NHX1_R	GAATGGCACCTAATGATATAATGGC
	SlNHX2	Sl_NHX2_F	TATCATTCGGTGCTACTAGCATTT
		Sl_NHX2_R	GATTGCTCCAATAGCAAGGTAATC
	SlSOS1	Sl_SOS1_F	CCGTATCTGGGCAAACATTG
		Sl_SOS1_R	CTGCACACCTCTTTATCTGGT
	SlSOS2	Sl_SOS2_F	GAGATTTGAAGCCTGAAAATTTGC
		Sl_SOS2_R	TCGACTCCTTGTTGAGGC
	SlSOS3	Sl_SOS3_F	GAGACCGCTTTTACGGTGAG
		Sl_SOS3_R	GCTGAAATTCTTCCTTGTGAATTAGT

	SlALMT9	Sl_ALMT9_F	CAGTATTGGAGCCACTCTAAGC
		Sl_ALMT9_R	CTTCCCATTCTCCAGCCAAT
	SlCCC	Sl_CCC_F	TGGAGCAATGTATGTATTGGGA
		Sl_CCC_R	GTGACAGTTTCTCTAAGAATTCCAG
	SlCLCc	Sl_CLCc_F	GCCTCGACTATGATATTGTAGAAAATG
		Sl_CLCc_R	AGAGCACAAGAGTCCATTTAAGA
	SlCLCg	Sl_CLCg_F	GCGAGAAGGTATACAACGGC
		Sl_CLCg_R	CAGCCTCTGGTGCGATAAA
	SlSLAH3	Sl_SLAH3_F	CCTGTCGTGGTAACTGAAGC
		Sl_SLAH3_R	GAGTAACCAGCCAAAGGAGAG
	SlACT	Sl_ACTIN_F	GAAATAGCATAAGATGGCAGACG
		Sl_ACTIN_R	ATACCCACCATCACACCAGTAT
	SlPP2Acs	Sl_PP2Acs_F	CGATGTGTGATCTCCTATGGTC
		Sl_PP2Acs_R	AAGCTGATGGGCTCTAGAAATC
	SlRPL2	Sl_RPL2_F	GTCATCCTTTCAGGTACAAGCA
		Sl_RPL2_R	CGTTACAAACAACAGCTCCTTC
Pepper	*CaAKT1*	Ca_AKT1_F	CCTCAACTATTTACAGTACGAACGA
		Ca_AKT1_R	ATTATTCATGATTATAGTCCCGTCCC
	CaAVP1	Ca_AVP1_F	GTCAGCTCTGTCGGCATC
		Ca_AVP1_R	GTTGCTTCTTCAATGCTGGC
	CaNHX1	Ca_NHX1_F	TGCAGGGTTTCAGGTAAAAAAG
		Ca_NHX1_R	AAAGGCACCTAATGTTATAATTGCA
	CaNHX2	Ca_NHX2_F	TCATTCTTCCGCAATTTCAGC
		Ca_NHX2_R	GAAAATGCCAATAGCACATAAAGATATG
	CaSOS1	Ca_SOS1_F	GTTGCTTGGTGGGCTTCTA
		Ca_SOS1_R	CATCAATGACTCGCCTTCGA
	CaSOS2	Ca_SOS2_F	CCTCAGAATCGTTGGGTCTTAA
		Ca_SOS2_R	GCAAATTGACCGGCCCTA
	CaSOS3	Ca_SOS3_F	GAGTGATGGAAAGATAGATCAAGATGA
		Ca_SOS3_R	GCTAAAGTAATATCCTTTAGATATGGAAGG
	CaALMT9	Ca_ALMT9_F	CATAACAGGTTTTTTTATCACTTATGCAA
		Ca_ALMT9_R	AACCGGATACCATGATGAAACA
	CaCCC	Ca_CCC_F	GCTGAAGTTATCGTCATTTCAATGA
		Ca_CCC_R	CTAACAACTCTTCGTTGTGCAG
	CaCLCc	Ca_CLCc_F	GACAAATTCAGAGAGTGGTGGTA
		Ca_CLCc_R	GTAAACGTTGGCTCCAACAATAG
	CaCLCg	Ca_CLCg_F	GTACCTGCTGGATATAATGCCT
		Ca_CLCg_R	TCCACTGTGTCTCTATGGTCTA
	CaCaAH3	Ca_SLAH3_F	AGCAAGCTGAAGGATAAAAGATTTG
		Ca_SLAH3_R	ACGGACTCTTGTTCTATTTCCTG
	CaACT	Ca_Actin_F	CCCAGATTATGTTTGAGACC
		Ca_Actin_R	GCAAAGCATAACCCTCATAG
	CaACT1	Ca_Actin1_F	GTCCTCTTCCAACCATCCAT
		Ca_Actin1_R	TACTTTCTCTCTGGTGGTGC
	CaGAPDH	Ca_GAPDH_F	ACCGCAACCCCATCAATCT
		Ca_GAPDH_R	CCAGTGTAGGAGTGTGTAG
